# Identification of loci controlling adaptation in Chinese soya bean landraces via a combination of conventional and bioclimatic GWAS

**DOI:** 10.1111/pbi.13206

**Published:** 2019-07-24

**Authors:** Ying‐hui Li, Delin Li, Yong‐qing Jiao, James C. Schnable, Yan‐fei Li, Hui‐hui Li, Huai‐zhu Chen, Hui‐long Hong, Ting Zhang, Bin Liu, Zhang‐xiong Liu, Qing‐bo You, Yu Tian, Yong Guo, Rong‐xia Guan, Li‐juan Zhang, Ru‐zhen Chang, Zhiwu Zhang, Jochen Reif, Xin‐an Zhou, Patrick S. Schnable, Li‐juan Qiu

**Affiliations:** ^1^ The National Key Facility for Crop Gene Resources and Genetic Improvement (NFCRI)/Key Lab of Germplasm Utilization (MOA) Institute of Crop Sciences Chinese Academy of Agricultural Sciences Beijing China; ^2^ Data Biotech (Beijing) Co., Ltd. Beijing China; ^3^ Department of Plant Genetics and Breeding China Agricultural University Beijing China; ^4^ Key Laboratory of Oil Crop Biology (MOA) Oil Crops Research Institute of Chinese Academy of Agriculture Sciences Wuhan China; ^5^ Departmentof Agronomy and Horticulture University of Nebraska‐Lincoln Lincoln NE USA; ^6^ Data2Bio LLC Ames IA USA; ^7^ Guangxi Academy of Agricultural Sciences Nanning China; ^8^ Department of Crop and Soil Sciences Washington State University Pullman WA USA; ^9^ Department of Breeding Research Leibniz Institute of Plant Genetics and Crop Plant Research (IPK) Gatersleben Germany; ^10^ Department of Agronomy Iowa State University Ames IA USA

**Keywords:** soya bean landrace, flowering time, bioclimatic variable, adaptation, associated SNP

## Abstract

Landraces often contain genetic diversity that has been lost in modern cultivars, including alleles that confer enhanced local adaptation. To comprehensively identify loci associated with adaptive traits in soya bean landraces, for example flowering time, a population of 1938 diverse landraces and 97 accessions of the wild progenitor of cultivated soya bean, *Glycine soja* was genotyped using tGBS^®^. Based on 99 085 high‐quality SNPs, landraces were classified into three sub‐populations which exhibit geographical genetic differentiation. Clustering was inferred from STRUCTURE, principal component analyses and neighbour‐joining tree analyses. Using phenotypic data collected at two locations separated by 10 degrees of latitude, 17 trait‐associated SNPs (TASs) for flowering time were identified, including a stable locus Chr12:5914898 and previously undetected candidate QTL/genes for flowering time in the vicinity of the previously cloned flowering genes, *E1* and *E2*. Using passport data associated with the collection sites of the landraces, 27 SNPs associated with adaptation to three bioclimatic variables (temperature, daylength, and precipitation) were identified. A series of candidate flowering genes were detected within linkage disequilibrium (LD) blocks surrounding 12 bioclimatic TASs. Nine of these TASs exhibit significant differences in flowering time between alleles within one or more of the three individual sub‐populations. Signals of selection during domestication and/or subsequent landrace diversification and adaptation were detected at 38 of the 44 flowering and bioclimatic TASs. Hence, this study lays the groundwork to begin breeding for novel environments predicted to arise following global climate change.

## Introduction

The widespread adoption of elite crop cultivars has resulted in substantial increases in productivity. Adaptive differences among crop cultivars are prime examples of the impact of natural and artificial selection on genetic polymorphisms. Therefore, one of the abiding goals of evolutionary genetics is to develop a better understanding of the genetic regulation of crop's environmental adaptation by identifying the genomic and geographic extent of adaptation. Two approaches that have been used in other species to link genomic regions or loci to local adaptation include genome‐wide association studies (GWAS) to identify genes controlling variation in specific adaptive traits (Fournier‐Level *et al*., 2011; Hancock *et al*., 2011) and reverse ecological approaches that use genome‐wide scans (GWS) to identify genes that exhibit significant differences in allelic frequencies indicative of selection among populations adapted to different environments (Beaumont and Balding, 2004; Günther and Coop, 2013). The wild progenitor (*Glycine soja* Sieb.&Zucc.) of cultivated soya bean (*Glycine max* (L.) Merr.) is one potential source of alleles conferring adaption to new environments (Hymowitz and Newell, 1981; Li *et al*., 2013). *G. soja* is naturally distributed throughout East Asia, including China, Korea, Japan and the far eastern regions of Russia, ranged from 24 to 53°N (latitude), 97 to 143°E (longitude) and 0 to 2650 m above sea level (altitude) (Xu *et al*., 1989). However, adaptive alleles in wild accessions are often linked with other loci that can confer undesirable agronomic traits (McNally *et al*., 2009). Soya bean was domesticated from *G. soja* in a region of China located at 35–40°N (Xu *et al*., 1986). Consequently, early landraces were not adapted to either high‐ or low‐latitude environments. Nonetheless, over hundreds of years of relatively stable climate, farmers developed many diverse landraces by selecting plants adapted to specific regions and to diverse cropping systems (Qiu *et al*., 2003; Zhou *et al*., 1998). These landraces are distributed across a broad ecogeographical range (18.2–51.4°N, 80.5–134.0°E) spanning a wide range of climactic conditions that differ with respect to sowing time, length of growing season, annual precipitation, maximum summer temperature, etc. (Chang and Sun, 1991; Chang *et al*., 1996; Wang, 1982). As a consequence of this selection history, these landraces exhibit a complex population structure (Li *et al*., 2008; Song *et al*., 2015; Zhou *et al*., 2015b). Moreover, improved cultivars exhibit 16%–25% less genetic diversity than the landraces, consistent with the view that landraces contain vast amounts of unexploited genetic diversity. Hence, these landraces may contain adaptive alleles (Hyten *et al*., 2006; Li *et al*., 2013; Zhou *et al*., 2015b). This untapped diversity could be used to adapt soya bean to future environments (Bandillo *et al*., 2017).

Flowering time is a major trait associated with soya bean adaptation, because soya beans are very sensitive to photoperiod. Hence, any given genotype is only adapted to a specific range of latitudes; a variety that flowers too early in a given environment will produce few seeds, while a variety that flowers too late will be killed by frost prior to seed production (Burgarella *et al*., 2016; Navarro *et al*., 2017). Several genes controlling flowering time, including *E1* to *E4*,* J*,* E9* (*FT2a*) and *FT5a* (Liu *et al*., 2008, 2018; Lu *et al*., 2017; Takeshima *et al*., 2016; Watanabe *et al*., 2009, 2011; Xia *et al*., 2012; Yue *et al*., 2017; Zhao *et al*., 2016) have been cloned. Further analysis of cultivars from different ecological zones indicated that the combinations of allelic variations for *E1* to *E4* and *J* determined the adaptation of soya bean to different latitudes (Jiang *et al*., 2014; Lu *et al*., 2017). However, we do not yet fully understand the genetic regulation of flowering time in soya bean (Tsubokura *et al*., 2014).

Here, we employed GWAS to identify loci that regulate flowering time in soya bean, as well as genes associated with local adaptation by genotyping a set of 1938 landraces and 97 *G. soja* accessions, and phenotyping these landraces in multiple environments and exploiting their GPS passport data to obtain high‐resolution historical weather data associated with the collection site of each landrace (Qiu *et al*., 2009, 2013; Wang *et al*., 2006). The identified ‘adaptive alleles’ could be used by breeders to develop new elite cultivars adapted to the environmental conditions expected to prevail in target regions at the date of release, as opposed to breeding cultivars adapted to environmental conditions as they exist today.

## Results

### Polymorphism, Population Structure and Diversity

**Figure 1:**
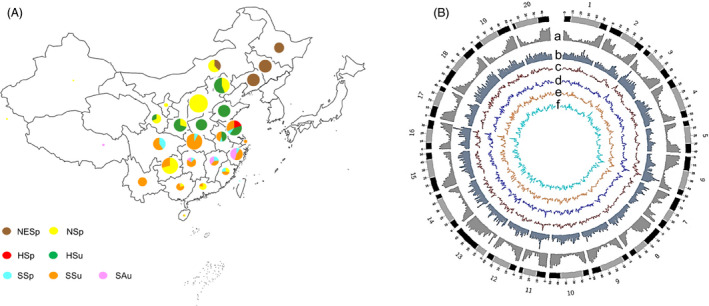
The geographical distribution of genotyped accessions and properties of the soya bean genome. The 2035 genotyped accessions include 1938 landrace and 97 wild soya bean accessions (A). Landraces were classified into seven ecotypes based on ecogeographical location and sowing time (Li *et al*., 2008; Zhou *et al*.,^1998^). The seven ecotypes included Northeast Spring‐type (NESp), North Spring‐type (NSp), Huang‐Huai Spring‐type (HSp), Summer‐type (HSu), South Spring‐type (SSp), Summer‐type (SSu) and Autumn‐type (SAu). A total of 99 085 SNPs were obtained across the 20 soya bean chromosomes as highlighted in the Circos plot (B). In the outer circle, heterochromatic regions are highlighted in grey and chromosome arms highlighted in black for the 20 soya bean chromosomes. (a) Gene density, (b) SNP density and (c‐f) genetic diversity (θw) of the ‘Wild’ (brown), ‘SR’ (dark blue), ‘HR’ (orange) and ‘NR’ (light blue) sub‐population inferred from a STRUCTURE analysis, respectively.

We genotyped a total of 2368 soya bean accessions (Table S1), including 112 annual wild soya bean selected from China, Korea, Russia and Japan to represent the ecogeographical range of *G. soja* and 2256 Chinese landraces. The latter were selected to represent much of the phenotypic diversity and geographic distribution of the 18 780 soya bean landraces from the Chinese National Soybean GeneBank (CNSGB)(Qiu *et al*., 2009, 2013; Wang *et al*., 2006). A total of 0.75 terabases (Tb) of sequence data from 5.9 billion quality‐trimmed reads was generated via tGBS® (Ott *et al*., 2017; Table S2). After alignment to the reference genome (*Glycine max* Wm82.a2.v1, https://phytozome.jgi.doe.gov/), a total of 186 122 single nucleotide polymorphisms (SNPs) were identified. After imputation and filtering, 333 accessions with >80% missing data were removed. Following these procedures, 99 085 SNPs each with a minor allele frequency (MAF) ≥ 1% and heterozygosity ≤20% (Figure S1) were available for 2035 accessions, including 97 *G. soja* and 1938 landraces (Figure 1A, Table S1). Each of SNPs is supported by an average of 13 sequencing reads per genotyped sample. These SNPs are well distributed across the genome (Figure 1B).

**Figure 2:**
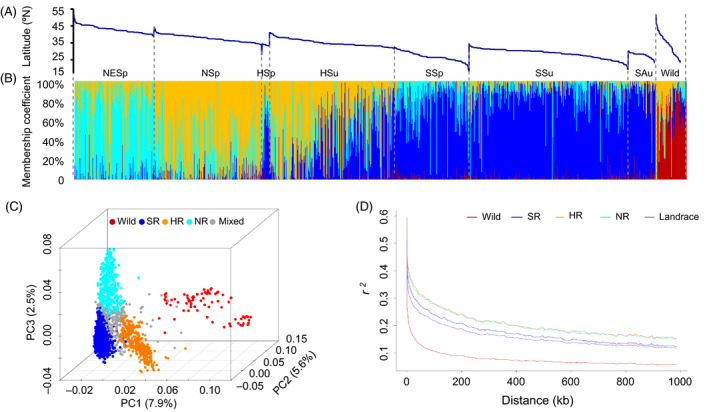
Population structure and linkage disequilibrium within sup‐populations. The samples are arranged by ecotypes and then by latitude of collection site (A). Structure analyses were conducted on the 1938 landrace and 97 wild accessions with *K *=* *4. The estimated proportions of an individual's membership in the corresponding populations are designated by percentages shown on the *y*‐axis (B). Principal component analysis (PCA) was conducted on 99 085 SNPs for the 1938 landrace and 97 wild accessions (C). The membership within samples was displayed as red for Wild, dark blue for Southern Region (NR), orange for Huanghuai Region (HR), light blue for Northern region (NR) and grey for Mixed. Linkage disequilibrium (LD) was calculated as *r* ^*2*^ (D). The decay of LD over distance is displayed within each sub‐population and all of the landraces combined (purple). Samples are labelled with the same colour scheme used for the PCA and LD plots (B‐C).

STRUCTURE analyses (Figures 2A,B and S2), principal component analyses (PCA; Figure 2C) and a neighbour‐joining tree (Figure S3) for the 2035 accessions identified one wild (termed ‘Wild’, *N* = 80) and three distinct sub‐populations of landraces. Based on the geographical origins of the accessions within each sub‐population (Table S3), they were termed ‘NR’ (Northern region, *N* = 385), ‘HR’ (Huanghuai region, from central China, *N* = 346) and ‘SR’ (Southern region, *N* = 1007). An additional 217 accessions with admixed genomes, labelled in grey in Figure 2C were classified as ‘Mixed’. Our results are consistent with a previous classification consisting of three main areas of soya bean production in China defined according to geographical distribution and are also in accordance with previously described scenarios of the history of soya bean differentiation (Li *et al*., 2008, 2010). In addition, these results are largely congruent with another prior classification system, defined based on ecogeographical location and sowing time (Gai and Wang, 2001).

### Population differentiation

Linkage disequilibrium (LD) plays a critical role in determining the precision with which causal loci can be identified via GWAS (Gupta *et al*., 2005). Variation in patterns of LD across the genome or between different populations can also provide information on the presence or absence of selective sweeps and selective pressure (Kim and Nielsen, 2004). The average extents of LD decay in the wild panel and the all‐landrace panel were 12 and 58 kb, respectively (Table S4). Although the number of accessions in the all‐landrace panel (*N* = 1938) was twenty times larger than the number of *G. soja* accessions (*N* = 97), the landraces still displayed more LD, fewer private SNPs and less diversity than the wild panel (Tables S4–S6).

Pairwise comparisons of the three landrace sub‐populations inferred from the STRUCTURE analysis exhibited different patterns of LD and different levels of diversity (Figure 2D, Table S5 and S6). NR and HR had similar patterns of LD decay, both of which were slower than the pattern of LD decay in SR. NR (θw = 1.08E‐05) exhibited less genetic diversity than HR (θw = 1.16E‐05) or SR (θw = 1.16E‐05). Pairwise Fst estimates between the three sub‐populations indicated the largest genetic differentiation was detected between HR and SR (0.164), followed by that between NR and SR (0.136), and the smallest differentiation was detected between NR and HR (0.077).

### Natural variation of bioclimatic variables and flowering time

The 1938 landraces were originally collected from sites ranging from 18.2 to 51.4°N latitude, from 82.5 to 132.6°E longitude and from 1 to 2520 m above sea level (Figures 1A and S4; Table S1). Moreover, these landraces were formed via selection for adaptation to specific regions and diverse cropping systems (Qiu *et al*., 2003; Zhou *et al*., 1998). As a result, the temperatures and photoperiods to which these landraces were originally adapted span a wide range of values. The temperature annual ranges (TARs) and maximum daylight lengths (MDLs) were calculated from sowing to harvest time in the collection locations and range from 14.4 to 58.7 °C and from 13.3 to 16.6 h (Figure S4). Annual precipitation (AP) ranges from 81 to 1986 mm in their collection locations. The distribution of precipitation reflects the topography of China, with a trend towards greater rainfall in the south.

The all‐landrace panel was grown and phenotyped in two locations, Beijing (40.1°N, 116.7°E) and Wuhan (30.5°N, 114.3°E). Substantial variation in flowering time among the accessions was observed at both locations. Flowering time ranged from 25.5 to 116 days in Beijing and from 23 to 78.5 days in Wuhan (Figure S4). The narrow‐sense heritability of flowering time was 0.625 in Beijing and 0.704 in Wuhan. Qst, a measure of phenotypic differentiation, was greater between pairwise combinations of landraces than Fst, a measure of genetic differentiation for phenotypic data from both Beijing and Wuhan (Table S7). This result is consistent with the divergence of flowering time across sub‐populations resulting from directional selection. Significant differences in flowering time were discovered across three inferred landrace sub‐populations in Beijing and Wuhan environments (Figure S5). While almost all of the landraces had shorter flowering times in Wuhan than in Beijing, the flowering times for each of the accessions at each of the two locations were significantly correlated (*r *=* *0.90, *P *<* *0.001). In combination, these results suggest that while absolute flowering time of a given landrace is quite plastic in response to environmental cues, variation in relative flowering time among landraces at a given growth location is primarily controlled by genetic factors.

### GWAS for flowering time

**Figure 3:**
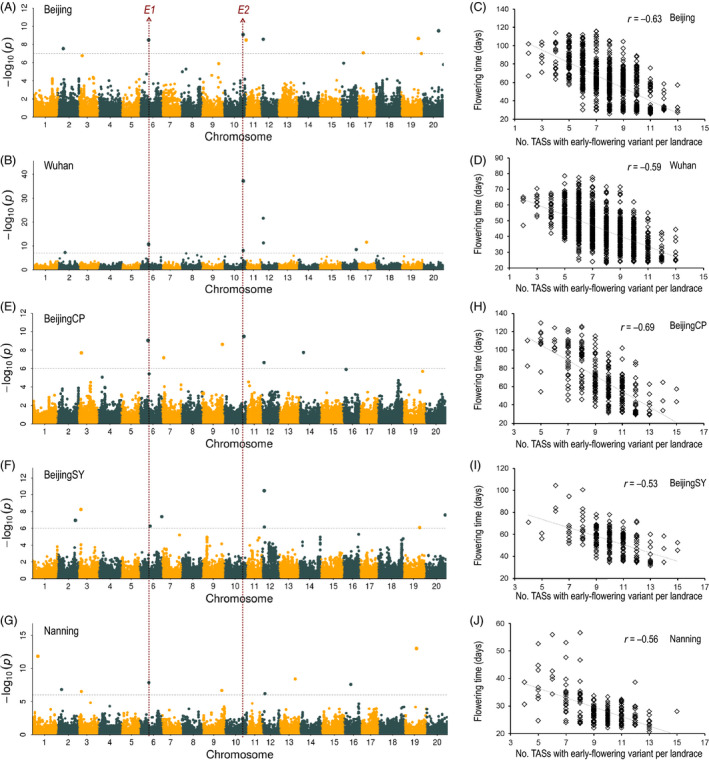
Identification and analysis of flowering time trait‐associated SNPs (TASs) from landraces. Manhattan plots from GWAS analyses for flowering time data collected from five locations, including Beijing (A), Wuhan (B), Beijing CP (E), Beijing SY (F) and Nanjing (G). Beijing and Wuhan plots are based on the all‐landrace panel (*N* = 1938); the other three plots are based on the core‐landrace panel (*N* = 414). Grey horizontal dashed lines indicate 1% Bonferroni‐corrected genome‐wide significance thresholds, 1.0E‐07 for Beijing and Wuhan, 1.0E‐06 for Beijing CP, Beijing SY and Nanjing. Red vertical lines designate the genomic locations of two cloned flowering time genes, *E1* and *E2*. Relationships between flowering times of landraces and the number of the 17 TASs that carry the early‐flowering variant across two locations for the all‐landrace panel Beijing (C) and Wuhan (D), and three locations for the core‐landrace panel, Beijing CP (H), Beijing SY (I) and Nanjing (J).

GWAS was conducted to identify genes associated with flowering time. Using a statistical significance cut‐off of *P *< 10^−7^(i.e. the 1% Bonferroni‐corrected threshold for 99 085 SNPs), a total of 18 associations were identified for flowering time across two locations separated by a 10° difference of latitude [Beijing (*N* = 9) and Wuhan (*N* = 9)] (Figures 3A,B and S6A,B, Table S8). The nine trait‐associated SNPs (TASs) identified from the Beijing location explained 58.0% of the phenotypic variance, and the nine TASs identified from the Wuhan location explained 57.5% of the phenotypic variance. These 18 associations represent 17 TASs because one SNP (Chr12:5914898) was associated with flowering time in both Beijing and Wuhan. Significant correlations were observed between the flowering times of members of the all‐landrace panel and the number of the 17 original TASs carrying early‐flowering alleles in a given landrace at both of these two locations (Figure 3C,D).

We also conducted GWAS on the core‐landrace panel (*N* = 414) using flowering time data collected from three sites (Beijing CP, Beijing SY and Nanning) over 4 years (2009–2012) (Figures 3E–G and S6C,D). Based on pairwise LD between identified SNPs (*r* ^2^ ≥ 0.8), four of 17 TASs identified in the all‐landrace panel were rediscovered (Table 1). Moreover, strong and significant correlations were observed between flowering times of members of the core‐landrace panel at the three environments and the number of the 17 original TASs carrying early‐flowering alleles in a given landrace (Figure 3H–J). These results validated the GWAS results from the all‐landrace panel.

**Table 1:** Common (based on LD blocks) flowering time TASs identified via five independent GWAS of two panels

TASs‐all	*P* value	MAF	PVE (%)	Location (No. of accessions)	Distance to the nearest cloned flowering gene	TASs‐core near TASs‐all					
						Position (bp)	Distance to TASs‐all (bp)	*r* ^2^ with TASs‐all	*P* value	MAF	Location
Chr06:20355903	3.2E‐09	0.269	12.4	Beijing (886)	148 kb to *E1* (Xia *et al*., 2012)	20 346 551	9352	0.820	1.4E‐08	0.366	Nanning
Chr10:45520960	7.0E‐38	0.412	16.5	Wuhan (1433)	204.8 kb to *E2* (Watanabe *et al*., 2011)	45 520 978	18	0.883	3.4E‐10	0.455	Beijing CP
Chr10:45521328	8.1E‐10	0.360	12.0	Beijing (886)	205.2 kb to *E2*(Watanabe *et al*., 2011)	45 520 978	350	0.883	3.4E‐10	0.455	Beijing CP
Chr12:5470311	2.6E‐22	0.124	0.9	Wuhan (1433)	–	5 496 042	25 731	0.938	2.3E‐07	0.221	Beijing CP

MAF, minor allele frequency; PVE, phenotypic variance explained; TASs core, TASs identified via GWAS of core‐landrace panel; TASs‐all, TASs identified via GWAS of the all‐landrace panel.

Thirteen of these 17 TASs were located within previously identified larger genomic intervals for flowering time QTLs (Table S8). We defined the LD block surrounding each these 17 TASs using *r* ^2^ ≥ 0.8 and examined these LD blocks for association signals and previously reported candidate genes. Ten of these 17 blocks contained flowering time TASs, and for five of these intervals, we identified previously described flowering time regulators of soya bean (including the two cloned soya bean flowering genes, *E1* and *E2*) or homologs of flowering‐time‐associated *Arabidopsis* genes (Table S8). Although we are not able to identify the specific causative gene responsible for most of the TASs, these results provide clear validation of the power of GWAS in this high LD species.

Based on the analyses of the observed differences in days to flowering between the two homozygous genotypes for the 17 flowering time TASs, we found that most (82.3%) of the 17 SNPs are associated with a consistent direction of effect on flowering time across the three landrace sub‐populations when phenotyped in the Beijing and Wuhan locations (Figure S7). Only Chr06:19873100 exhibited a reversed direction among sub‐population. NR and HR landraces carrying Chr06:19873100‐AA showed significant earlier flowering time than those carrying Chr06:19873100‐GG with an average of 12.1 days in Beijing and 3.1 days in Wuhan location. But their genotypic effect was reversed in the SR sub‐population. Further analyses of the effect of Chr06:19873100 among spring sowing type, summer sowing type and autumn sowing type landraces in the SR sub‐population indicated that the direction of effect of Chr06:19873100 in the NR and HR sub‐populations was consistent with that in the spring sowing type landraces within the SR sub‐populations, but was reversed in the summer sowing type landraces within SR sub‐populations (Figure S8). This phenomenon may reflect different effects of the same locus under the different natural conditions that vary with respect to photoperiod, temperature, and other environmental parameters and crop management practices.

### GWAS for bioclimatic parameters

We used GWAS to identify loci associated with adaptation to three bioclimatic variables (temperature/TAR, daylength/MDL and precipitation/AP) that we treated as quantitative traits based on the location of the collection site of each accession. A total of 29 significant association signals (*P *<* *10^−7^) for TAR (7 SNPs), MDL (16 SNPs) and AP (6 SNPs) corresponding to 27 unique TASs (Figure S9, Table S9) were detected. Consistent with our observation of strong correlations between MDL and TAR (*r* = 0.96, Figure S4), two SNPs (Chr02:6487107 and Chr15:23361474) were associated with both MDL and TAR.

In accordance with the observation that all of three bioclimatic variables (TAR, MDL and AP) are tightly correlated with flowering times in soya bean (Figure S4), we identified a series of soya bean flowering genes (*E1*,* GmFT2c*,* GmELF4*, etc.) or homologs of flowering‐time‐associated *Arabidopsis* genes (Table S9) in the LD blocks surrounding 12 of the bioclimatic TASs. The most significant MDL TAS Chr06:20055100 (*P* value = 2.4E‐17) is located within the LD block that contains the *E1* locus. Of 146 landraces carrying Chr06:20055100_AA, 83.6% were from high‐latitude NR cluster. In the NR cluster, landraces with Chr06:20055100_AA were significantly earlier flowering than those that carried Chr06:20055100_GG (*p*of *t*‐test = 5.0E‐20 for Beijing and 7.3E‐24 in Wuhan locations). The most significant AP TAS Chr18:2029602 (*P* value = 1.9E‐13) was located 34 kb away from the flowering gene *GmELF4* (Marcolino‐Gomes *et al*., 2017). In addition, we detected *Glyma.15G196500*, the homolog of *phyE* which plays an important role in the integration of flowering, light and temperature cues (Sánchez‐Lamas *et al*., 2016) located in the LD block surrounding MDL and TAR‐associated TAS Chr15:23361474. More interestingly, nine sub‐population‐specific (or with particularly low MAF (<5%) in one and/or two populations) alleles of bioclimatic TASs showed significant differences in flowering time in these corresponding sub‐populations (Figure S10). This suggests that the identification of bioclimatic TASs via GWAS has the potential to help elucidate the genomic basis of variation in flowering time.

### Detection of selection signals during soya bean domestication and the diversification of landraces during adaptation

The diversity of soya bean landraces is mainly derived from the annual wild soya bean, *G. soja*. We estimated the genetic differentiation of these flowering time and bioclimatic TASs using Fst and allele frequency comparisons between landraces and *G. soja*. We found evidence that 52.9% of the 17 flowering time TASs and 18.5% for the 27 bioclimatic TASs experienced strong selection during domestication (Table S10). The frequency of the accessions containing early‐flowering alleles of the five flowering time TASs (Chr02:12358355‐TT, Chr06:20003061‐TT, Chr12:5470311‐TT, Chr12:5914898‐CC and Chr17:20231663‐AA) increased after domestication. This finding is consistent with the hypothesis that the early flowering trait was selected during domestication.

Directed selection resulting from human needs and geographical adaptation is expected to strengthen population differentiation at the genomic regions that are the targets of selection. Significant differences in flowering time, MDL, TAR and AP observed among north (NR), central (HR) and south (SR) China, encouraged us to further investigate the geographic differentiation of these TAS alleles. By comparing Fst and allele frequency among pairwise sub‐populations of landraces, we detected 35 (12 flowering time and 23 bioclimatic) adaptation‐related TASs exhibiting various differentiation patterns among three sub‐populations of landraces (Figure S11 and S12, Table S11). For seven of the 17 flowering time TASs and 20 of the 27 bioclimatic TASs, the minor allele was found only in geographically constrained sets of landraces or exhibited low MAF (<0.05) in one or two sub‐populations (Figure S11 and S12, Table S11). Of these 27 sub‐population specific TASs, ten were specific to NR, two were specific to HR, eight were specific to SR, one was specific to NR and HR, three were specific to NR and SR.

Of the 17 flowering time and 27 bioclimatic TASs, 11 experienced selection during both domestication and subsequent landrace diversification and adaptation; three underwent selection only during domestication; 24 underwent selection only during landrace diversification and adaptation; and six did not exhibit signals of selection (Tables S10 and S11). These analyses results provide evidence for natural and/or artificial selection for adaptation to specific environment conditions across China and provide an initial picture on how bioclimatic variables shaped patterns of genetic variation among soya bean landraces.

### Identification of candidate flowering QTL/genes

*E1* is a novel transcription factor containing a B3‐related domain, which has a large effect on flowering time (Cao *et al*., 2016; Zhai *et al*., 2015). We found three TASs (Chr06:19873100, Chr06:20003061 and Chr06:20355903) near the*E1* gene on Chr06. These three TASs cover a 482.8 kb region, but belong to different LD blocks on the basis of pairwise LD correlations (*r* ^2^ ≥ 0.8). The alleles of Chr06:19873100, Chr06:20003061 and Chr06:20355903 had different effects on flowering time, which explained 15.0% (Wuhan location), 8.4% (Wuhan location) and 12.4% (Beijing location) of phenotypic variance, respectively. They also had different patterns of geographical distribution (Figure S11). For example, the late‐flowering genotype (CC) of Chr06:20003061 was present at a frequency of 5.7%, and landraces carrying Chr06:20003061‐CC were collected from low‐latitude regions (south of 34°N). In contrast, the early‐flowering genotype (CC) of Chr06:20355903 was present at a frequency of 27.3%, and the landraces carrying it were mainly collected from the central and north of China (Figure S11), similar to *E1* (Zhou *et al*., 2015b). In combination, these results suggest that there may be one or more previously undetected flowering time‐related loci near *E1*.

The *E2* gene, an orthologue of *Arabidopsis* circadian clock‐controlled gene *GIGANTEA*, was tagged by Chr10:45375315, which was significantly associated with flowering time measured at the Wuhan location. The landraces with Chr10:45375315‐GG genotype exhibited 11.1 and 6.6 days earlier flowering time than those landraces with Chr10:45375315‐TT genotypes in the Beijing and Wuhan locations, respectively. Only 65 landraces carried the early genotype Chr10:45375315‐GG, which was mainly (90.8%) collected in China north of 33°N. In addition to Chr10:45375315, two other TASs were detected near *E2*. Chr10:45520960 had the strongest association signal and explained the largest proportion of phenotypic variance (16.5%) in the Wuhan environment. Chr10:45521328 had the second strongest association signal and explained the second largest proportion of phenotypic variance (12.0%) in the Beijing location; these two TASs are located 204.8 kb and 205.2 kb away from *E2*, respectively, but only 369 bp away from each other. Pairwise LD correlations using *r* ^2^ ≥ 0.8 suggest that these two TASs are located within a candidate region from 45 426 954 to 45 554 899 bp, which overlaps a previously reported QTL *First flower 24‐4* (from soybase.org) 55.8 kb away from the bloom date‐related locus Chr10_45465189 (Fang *et al*., 2017). Of three genotypic combinations formed by Chr10:45520960 and Chr10:45521328, most landraces carry Chr10:45520960‐AA/Chr10:45520960‐GG or Chr10:45520960‐GG/Chr10:45520960‐AA (57.6% and 42.4%, respectively), while few carry Chr10:45520960‐AA/Chr10:45520960‐AA (0.1%). Landraces that carry Chr10:45520960‐AA/Chr10:45520960‐GG flowered 19.5 and 9.8 days earlier than those that carry Chr10:45520960‐GG/Chr10:45520960‐AA in Beijing and Wuhan locations, respectively. In the NR sub‐population, 95.1% of landraces carried the early‐flowering Chr10:45520960‐AA/Chr10:45520960‐GG genotype. Moreover, a significant (*P *<* *2.2e‐16) difference in flowering time was detected in the NR sub‐population between the landraces carrying these two genotypes (Figure S13). These results pointed to *Glyma.10G224500* being a candidate flowering time‐related gene.

**Figure 4:**
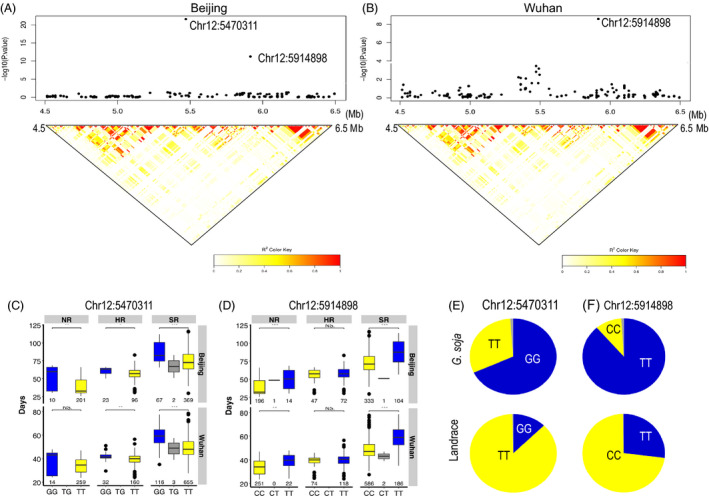
Identification of Chr12:5470311 and Chr12:5914898 flowering time loci. (A) Local Manhattan plots and LD heatmaps (Shin *et al*. 2006) surrounding Chr12:5470311 and Chr12:5914898 in the Beijing (A) and Wuhan locations (B). Boxplots for flowering times in three defined landrace sub‐populations based on the genotypes for Chr12:5470311 (C) and Chr12:5914898 (D). Box edges represent the 0.25 and 0.75 quantiles with median values shown by bold lines. Genotype frequencies at Chr12:5470311 (E) and Chr12:5914898 (F) in the wild panel (*G. soja*) and the all‐landrace panel.

In addition, two flowering time TASs on chromosome 12 (Chr12:5470311 and Chr12:5914898) were identified via GWAS using experimentally measured flowering time data from the Beijing and Wuhan locations. Chr12:5470311*,* which explained 0.9% of the phenotypic variance, was the second strongest signal for association with flowering time in Wuhan, while Chr12:5914898 exhibited a significant association with flowering time at both locations and explained 1.8% and 2.3% of phenotypic variance in Wuhan and Beijing, respectively (Figure 4A,B, Table S8). The 92.9 kb LD block surrounding Chr12:5470311 contains the candidate flowering gene (*Glyma.12G073900, GmPRR3a*), a homolog of the *Arabidopsis* flowering gene *PSEUDO‐RESPONSE REGULATORS 3* (*AtPRR3*) (Para *et al*., 2007). Chr12:5470311 exhibits LD (*r* ^2^ = 0.938) with the SNP marker Chr12:5496042, which was identified as a flowering time TAS (*P *=* *2.3E‐07) in the core‐landrace panel in Beijing CP location (Table 1). Chr12:5914898 is located in an intron of the *Glyma.12G076800* gene (*Glyma12 g08160* in v1.1), which encodes a Cyclic Nucleotide‐gated Ion Channel 15‐Related protein. The consistency of the associations was tested by comparing the flowering times of particular genotypes of Chr12:5470311 and Chr12:5914898 SNP sites within each of the three landrace sub‐populations defined in this study (Figure 4C,D). Accessions that carry Chr12:5470311‐GG and/or Chr12:5914898‐TT genotypes flowered significantly later within each of the three sub‐populations than did genotypes homozygous for the alternate alleles.

Fst and allele frequency analysis demonstrated that Chr12:5470311 and Chr12:5914898 underwent selection during soya bean domestication (Figure 4E). Chr12:5914898 is adjacent to a reported domestication region (5 903 254–5 907 102 bp)(Zhou *et al*., 2015a). During the breeding of early‐flowering soya beans, the frequency of the unfavourable late‐flowering genotypes Chr12:5470311‐GG and/or Chr12:5914898‐TT decreased from 0.686 in *G. soja* to 0.127 in landraces and from 0.892 *G. soja* to 0.271 landrace, respectively. After domestication, both alleles of Chr12:5470311 were distributed across China, from the south to the northeast region (Figure S14A), whereas the distribution of Chr12:5914898 alleles reflects genetic differentiation between the HR sub‐population and the two other sub‐populations (NR and SR) (Figure S14B).

## Discussion

China maintains 70% of the unique soya bean accessions in gene banks worldwide, and many of these accessions are genetically diverse and ancient landraces (Qiu *et al*., 2011). There are at least two challenges in utilizing such germplasm collections for crop improvement. First, simply phenotyping large numbers of individuals, particularly in multiple environments, is both time and resource intensive. Second, it is particularly difficult to collect equivalent measurements from accessions that exhibit extensive physiological and morphological variation, as is often the case with landrace accessions. In this study, 1938 representative landraces, selected to represent much of the phenotypic and geographic diversity of the Chinese collection, were used to identify SNPs associated with both empirically measured flowering time across multiple environments and bioclimatic parameters extracted from passport data associated with the collection sites of accessions.

We identified 18 significant (*P* < 10^−7^) association signals (tagged by 17 TASs) with flowering time across two geographic locations. Surprisingly, only one of these TASs was identified at both locations. Although it has been reported that soya bean flowering is controlled by both environment‐insensitive and environment‐sensitive loci and the latter type is in the majority (Mao *et al*., 2017), the findings that effect sizes and directions for most SNPs were well conserved across both locations and that the rank orders of landrace flower times were well conserved across the two locations suggest that flowering time in soya bean exhibits little GxE. It is therefore likely that the substantial differences in the compositions of the panels analysed at the two locations are responsible for why different TASs were detected at the two locations. For example, as compared to the Wuhan GWAS panel (*n* = 1433), 547 landraces collected from South China were excluded from the Beijing GWAS panel (*n* = 886) because they are not adapted to flower properly in Beijing.

The identified flowering time SNPs included both some that are near genes previously associated with flowering time, as well as some newly identified associations. This conclusion is based on the fact that we identified more than one candidate QTL/gene region near the cloned flowering gene *E1* as well as more than one candidate QTL/gene region near the *E2* gene. Because population structure can cause false‐positive GWAS hits (Larsson *et al*., 2013), we controlled for it during our GWAS. Further, because soya bean populations are geographically stratified (Figure 2) (Li *et al*., 2010; Zhou *et al*., 2015b), it is probable that our control for population structure removed true positives and thereby inadvertently produced some false negatives. In addition, when two populations exhibit oppositely phased coupling linkage between a marker and a causative gene, their signals may cancel out in a combined GWAS. To a certain extent, these challenges were overcome by conducting GWAS on individual sub‐populations.

In addition to directly seeking to identify associations between SNPs and flowering time, passport data associated with the collection sites of the landrace accessions allowed us to identify SNPs associated with three bioclimatic parameters (viz., temperature, daylength and precipitation). Some of these SNPs exhibited associations with flowering time per se *within* individual sub‐populations. This could be a consequence of differential LD among populations (Figure 2C) and/or different linkage relationships between markers and causative alleles among sub‐populations. For example, within a population with slower LD decay (such as NR) it may be easier to detect associations between markers and causative genes even though mapping precision will be lower. Because a similar analysis reported for maize (Navarro *et al*., 2017) involving bioclimatic parameters successfully identified SNPs associated with flowering time, the challenges associated with population structure reported in the current study may be of particular importance in self‐pollinated species where LD decay is slower and greater population structure is expected.

New crop varieties are typically developed by evaluating their field performance. These field tests are necessarily conducted in current environments. Global climate change complicates this paradigm because the target environments for the new varieties may not yet exist. In the current study, using data about soya bean landraces developed via selection during centuries of relatively stable climate we were able to detect loci underlying adaptive flowering time variation. Many of these candidate genes are well supported by ancillary evidence. As such, this study lays the groundwork to begin breeding for novel environments predicted to arise as a consequence of global climate change.

## Plant materials and methods

### Plant materials

A total of 2368 soya bean accessions were analysed in this study; these accessions were from two unique germplasm diversity panels maintained at The Chinese Academy of Agricultural Sciences (CAAS, Table S1), including

The ‘wild panel’ consists of 112 annual wild soya bean accessions selected from China, Korea, Russia and Japan, and represents the ecogeographical range of *G. soja*. After discarding 15 accessions with ≥80% missing SNP data, the final wild panel included 97 *G. soja* accessions.

The ‘all‐landrace panel’ comprised 2256 landraces; these were originally collected from areas across China. Most of landraces (85.3%, 1925 accessions) were selected from a Chinese primary core collection that was initially developed to capture as much of the phenotypic diversity and geographic distribution of the collection of 18 780 cultivated soya beans present in the Chinese National Soybean GeneBank (CNSGB) as possible (Qiu *et al*., 2009; Wang *et al*., 2006). We also included 331 additional landraces that exhibited at least one ancestral phenotypic trait (*e.g*. small seed size, semi‐erect stem or seed bloom) but which were not present in the primary core collection to this all‐landrace panel. After discarding 318 accessions with ≥80% missing SNP data, the final all‐landrace panel included 1938 accessions. Note that, due to divergent farming and cropping systems, as well as climactic variation among soya bean growing regions, previous work has divided soya bean landraces into seven original ecotypes according to ecogeographical and planting season types, specifically: Northeast spring‐type (NESp), North spring‐type (NSp), Huang‐Huai spring‐type (HSp), Huang‐Huai summer‐type (HSu), South spring‐type (SSp), South summer‐type (SSu) and South autumn‐type (SAu) (Li *et al*., 2008; Zhou *et al*., 1998). Of the 1938 landraces examined in this study (after removing lines with ≥80% missing SNP data), 269 were classified as belonging to NESp, 362 from NSp, 26 from HSp, 415 from Hsu, 249 from SSp, 529 from SSu and 88 from SAu (Table S1).

We also analysed a ‘core‐landrace panel’, based on the Chinese core‐landrace panel that included 414 all‐landrace accessions, all of which are also present in the all‐landrace panel of the present study (Qiu *et al*., 2009, 2013; Wang *et al*., 2006).

### Phenotyping

The all‐landrace panel (2256 soya bean landraces) was planted and phenotyped at two locations: in early July 2015 in Wuhan city in Hubei province (30.5°N, 114.3°E), and in late June 2015 in Beijing city (40.1°N, 116.7°E) (Figure S10). Experiments were conducted using a completely randomized experimental design with two complete replicates, and the core‐landrace panel (414 landraces) was planted and phenotyped in three locations over several years. The three locations were as follows: Nanning city in Guangxi province (2009–2011), Changping District in Beijing (Beijing CP) and Shunyi District in Beijing (Beijing SY) (2011–2012). The landraces planted in Beijing CP were sown in early May (to mimic a spring growing season), in Beijing SY in late June (to mimic a summer growing season) and in Nanning in middle July. Flowering time and maturity time were scored across experiments. Flowering time was scored as the number of days from the emergence of the cotyledons to the appearance of flowers in 50% of the plants within the row, and maturity time was defined as the number of days from the date of emergence of the cotyledons to the appearance of maturity (95% of pods coloured brown) in 50% of the plants within the row (Qiu *et al*., 2006). For field trials with the all‐landrace panel, the flowering time of the two replicates was averaged for each landrace. For field trials with the core‐landrace panel, the averaged value for each accession in each location using 3 years and 2–3 replicates was used for final analysis.

### Ecogeographical and BioClimatic variables

The latitude, longitude and altitude data of the original collection locations of Chinese wild soya bean and landraces were obtained from previously published information (Chang and Sun, 1991; Chang *et al*., 1996; Li, 1990; Wang, 1982); those of wild soya bean from Japan, South Korea and Russia were obtained from the Germplasm Resources Information Network (http://www.ars-grin.gov/). The latitude and longitude coordinates of wild soya bean and landraces were used to query two bioclimatic variables: temperature annual range (TAR, BIO7) and annual precipitation (AP, BIO12) from BioClim (http://www.worldclim.org/bioclim, version 1.4), using the highest resolution dataset presently available (30 arc‐seconds (~1 km), 0.93 × 0.93 = 0.86 km^2^ at the equator) (Hijmans *et al*., 2005). Maximum daylight length observed between sowing and harvest time in the original locations of landraces (MDL) was calculated as previously described (Teets, 2003).

### DNA extraction, sequencing and data trimming and alignment

Young leaf tissue was collected from each accession, and total genomic DNA was isolated using the CTAB method, as described previously (Xie *et al*., 2005). Tunable genotyping by sequencing (tGBS®), with one base pair of selectivity, was performed and sequenced on Life Technologies’ Ion Proton Systems by Data2Bio LLC, as previously described (Ott *et al*., 2017).

Each individual sequence read was scanned and trimmed for regions of low‐quality sequence (defined as having a PHRED quality score <15). Trimming was conducted in two phases: (i) low‐quality nucleotides of each read end were removed; (ii) remaining nucleotides were then scanned using overlapping windows of 10 bp, and sequences beyond the last window with average quality value less than PHRED 15 were truncated. Trimmed reads were aligned to the *Glycine max* Wm82.a2.v1 reference genome using GSNAP (Wu and Nacu, 2010; Wu and Watanabe, 2005). Subsequently, confidently mapped reads were filtered if it mapped uniquely (≤2 mismatches every 36 bp and <5 bases for every 75 bp as tails), and used for subsequent analyses.

### SNP calling, genotyping and imputation

Polymorphic sites with alleles which differ from the reference genome were identified within each soya bean accession. While doing so, the first and last 3 bp of each read was ignored; only sites with PHRED quality ≥20 and which were covered with at least five reads were considered; only bi‐allele sites with a combined overall allele frequency of ≥80% were retained as polymorphic sites.

The genotypes of all retained bi‐allele polymorphic sites were determined for each accession. Homozygous SNP sites were defined as having ≥2 reads of major allele, and overall major allele reads account ≥90%. Heterozygous SNP sites were defined as having ≥1 read for each of two alleles, each allele accounting for 20% of total reads. Meanwhile, the sum of those two alleles should be at least five reads and account for ≥90% of total reads. For all other situations, a missing genotype was assigned.

Genotyping filtering criteria were applied in R 3.3.2 (R Core Team, 2016) to improve quality of polymorphic sites data. First of all, we required a SNP to have a minor allele frequency (MAF) of ≥1%, heterozygous rate ≤20% and a missing data rate ≤70% among all polymorphic sites; among remaining SNPs, we removed 333 samples with a missing data rate ≥80%. Next, imputation was performed among the remaining SNPs and samples using Beagle (V4.1) and default parameters without a reference panel (Browning and Browning, 2007, 2016). Finally, in addition to applying the aforementioned filtering criteria, imputed SNPs were also required to be present as a homozygous minor allele in ≥20 soya bean accessions. In the end a high‐quality SNP set, consisting of 99 085 SNPs was obtained.

### Population structure

Population structure was characterized using three methods. (i) a model‐based clustering approach implemented in the software package STRUCTURE (Falush *et al*., 2007; Pritchard *et al*., 2000) using ‘BURNIN 100000’ and ‘NUMREPS 100000’ with other default parameters. To clarify the hierarchical population structure in this diversity panel, the analysis was initially conducted with all samples. Then, population structure was identified by further separately analysing the distinct populations identified in the first step. The number of populations (*K*) was assessed from 1 to 10 for all of analysis. And best *K* was inferred using the ‘Evanno’ method (Evanno *et al*., 2005) implemented in STRUCTURE HARVESTER (Earl, 2012). We filtered the imputed 99 085 SNPs, which has ≥1% MAF among all samples, among each subset samples with a ≥MAF 5%. After that 10 000 SNPs were randomly selected for STRUCTURE analyses. (ii) Pairwise distances were estimated between soya bean accession using an unbiased model of substitution frequencies on those 99 085 SNPs. Distance estimates were then used to construct a phylogenetic tree using the neighbour‐joining‐like algorithm implemented in the APE R package (V3.2) (Paradis *et al*., 2004) Then, the tree was visualized and customized with tools on the EvolView website (http://www.evolgenius.info/evolview/) (He *et al*., 2016). (iii) Principal component analysis (PCA) was performed based on the 99 085 SNPs based using a variance‐standardized relationship matrix implemented in PLINK (V1.9) (Purcell *et al*., 2007).

Weir and Cockerham's fixation index (Fst) (Weir and Cockerham, 1984) was calculated by VCFtools (Danecek *et al*., 2011) for pairwise sub‐populations on a per SNP basis and sliding window (30‐kb window) separately.

### Gene diversity

The gene diversity, that is Watterson's estimators of theta (θw), was evaluated using software package VariScan (V2.0.3) (Hutter *et al*., 2006) among the whole population and each sub‐populations, respectively. SNPs with a MAF of ≥1% within each set of samples were used to characterize a given set of samples. Sliding window with a 1 Mb window length was used to scan the whole genome. The central 95% range and average value were calculated among θw of each window to present polymorphism.

### Qst‐Fst comparisons

Pst, a reasonable Qst proxy (Leinonen *et al*., 2006, 2013), was used to assess phenotypic differentiation among pairwise sub‐populations for given traits. An R package, Pstat (V1.2) (Silva and Silva, 2018) was used to calculate Pst with a bootstrap of 1000 along a 95% confidence level interval. Mean of Weir and Cockerham's Fst between pairwise sub‐populations was used to compare with the corresponding Pst.

### Linkage disequilibrium (LD)

To evaluate LD within wild, landrace and the landrace sub‐populations, PLINK (V1.9)(Purcell *et al*., 2007) was used to estimate the correlation (*r* ^2^). This calculation was made for all possible pairs of SNPs with MAF ≥1% in the target population, which were separated by 1 megabase or less. Average LD values were calculated using all pairs of SNPs within 1‐kb windows of distance beginning at 0–1 kb and ending at 999–1000 kb. The reported LD distance was defined as the distance at which the average *r* ^2^ between SNPs declined to one‐half of its maximum observed value.

The pairwise *r* ^2^ was calculated between trait‐associated SNPs (TAS) and its two sides 1 Mb‐context SNPs; the LD block region of TAS was defined by its farthest 1 Mb‐context SNPs with a *r* ^2^ ≥ 0.8 in each side.

### Heritability

The narrow‐sense heritability [$h^{2}=\sigma_{a}^{2}/\left(\sigma_{a}^{2}+\sigma_{e}^{2}\right)$] was assessed from the variance component estimates of the mixed models implemented in GAPIT (Lipka *et al*., 2012; Zhang *et al*., 2010). $\sigma_{a}^{2}$ is the estimated additive genetic variance and $\sigma_{e}^{2}$ is the residual effect.

### Genome‐wide association study (GWAS)

Genome‐wide association study of the all‐landrace panel and the core‐landrace panel was conducted using the Fixed and random model Circulating Probability Unification (FarmCPU) package (Liu *et al*., 2016). For the all‐landrace panel, GWAS was conducted across eight variables including three ecogeographical (latitude, longitude and altitude), three climate (illumination hours, precipitation and temperature) and one phenology variable, flowering time in Beijing and Wuhan. Corresponding to a ‘break’ on the *P*‐value distribution as represented by a QQ‐plot for most variables, the threshold for significant association was determined as 10^−7^ (≈0.01/No. of SNPs) in each association analysis. For the core‐landrace panel, GWAS was conducted for flowering time in Beijing CP, Beijing SY and Nanning. Considering the smaller number of SNPs decreased by population size of the core‐landrace panel, the threshold for significant association was determined by 10^−6^.

### Phenotypic variance explained (PVE)

To evaluate the phenotypic variance explained by each TASs, a full model was constructed to include all the identified SNPs, that is $Y=\mu +\sum_{i=1}^{q}\beta_{i}x_{i}$, where *Y* is the observed phenotype, μ is the mean performance, *q* is the number of identified SNPs, β_*i*_ and *x*_*i*_ are the effect and genotype of each identified SNP. The coefficient of determination $R_{\mathrm{full}}^{2}$ can be obtained from the full model. A reduced model was constructed to exclude one SNP at a time, say the *j* ^th^ SNP, that is $Y=\mu +\sum_{i\neq j}^{q}\beta_{i}x_{i}$. Thus $R_{j}^{2}$ can be obtained as ${\text{PVE}}_{j}=R_{\mathrm{full}}^{2}-R_{j}^{2}$.

## Conflict of interest

The authors declare no conflicts of interest.

## Author contributions

Y‐H.L., J.C.S., P.S.S. and L.Q. conceived the study. Y‐H.L., J.C.S., Z.Z., J.R., P.S.S. and L.Q., jointly wrote the paper. Y‐H.L., Y‐F.L., Y.T. and L.Z. provided DNAs. Y.J., Y‐F.L., H.C., H.H., Z.L., Q.Y. Y.G. R.G. and X.Z. collected the phenotype data. Y‐H.L., D‐L.L., J.C.S., Y‐F.L., H‐H.L., T.Z., B.L., R.C. P.S.S. and L.Q. performed sequencing/SNP calling/population/evolutionary/biology analyses.

## Data deposition and accession numbers

All the DNA sequence data have been deposited into the Sequence Read Archive (SRA) under BioProject accession PRJNA454779, PRJNA454780 and PRJNA477242.

## Supporting information


**Figure S1** Summary of 99 085 imputed SNPs.
**Figure S2** Δ*K* values as a function of *K*, the number of putative sub‐clusters within 2035 soybean accessions.
**Figure S3** Neighbor‐joining tree of 2035 soybean accessions based on shared allele pairwise distances.
**Figure S4** Pearson's co‐efficient for pairwise comparison of eight variables, including flowering time in Beijing and Wuhan, three geographical, and three climate variables in soybean landraces.
**Figure S5** Variation in flowering time across three defined sub‐populations of soybean landrace at Beijing and Wuhan locations.
**Figure S6** Quantile‐quantile plots from GWAS analyses for flowering time data collected from five locations, including Beijing (A), Wuhan (B), Beijing CP (C), Beijing SY (D) and Nanjing (E).
**Figure S7** Genotype effects (measured as the difference in days between two homozygous genotypes) of the 17 flowering TASs in three sub‐populations of landraces at the Beijing and Wuhan locations.
**Figure S8** Boxplots for flowering times in three sowing types of SR sub‐population based on the different genotypes of Chr06:19873100 in Beijing and Wuhan locations.
**Figure S9** Manhattan and quantile‐quantile plots resulting from genome‐wide association studies for bioclimatic variables in soybean landraces.
Grey horizontal dashed lines indicate 1% Bonferroni‐corrected genome‐wide significance thresholds, 1.0E‐07.
**Figure S10** Genotype frequencies in three sub‐populations of landraces inferred from STRUCTURE analysis and the genetic effects on associated flowering times across two locations, Beijing and Wuhan, at nine bioclimatic TASs that are sub‐population‐specific (or with particularly low MAF (<5%) in one and/or two populations).
**Figure S11** Geographic distributions of genotypes in 1938 Chinese landraces of 17 SNPs associated with flowering time at Beijing and/or Wuhan locations. Blue dots indicate minor homozygous, yellow dots indicate major homozygous and grey dots indicate heterozygous genotypes.
**Figure S12** Geographic distributions of genotypes in 27 SNPs associated with bioclimatic variables.
**Figure S13** Boxplots for flowering times in three defined sub‐populations of landraces based on the two predominately genotypic combinations of Chr10:45520960 and Chr10:45521328.
**Figure S14** Distribution of genotypes of Chr12:5470311 and Chr12:5914898 in three Chinese sub‐populations of landraces.
**Table S3** No. of soybean landraces in pre‐defined species or cultivated ecotypes assigned into populations inferred from STRUCTURE analysis.
**Table S4** Summary statistics of soybean linkage disequilibrium (*r* ^2^) within different sub‐groups on accessions.
**Table S5** Summary statistics for genome‐wide SNPs and estimates of gene diversity (θw) across 2035 soybean accessions.
**Table S6** Population differentiations between *G. soja* and landraces and between pairs of four defined sub‐populations of landraces.
**Table S7** Evaluation of Pst by comparing mean Qst and Fst estimates for flowering times between pairs of sub‐populations of landraces.
**Table S8** 17 SNPs associated with flowering time in landraces measured at Beijing and Wuhan locations.
**Table S9** 27 TASs associated with three bioclimatic variables [temperature annual range (TAR), annual precipitation (AP) and maximum daylight length (MDL)].
**Table S10** Allelic status and Fst in 17 flowering time and 27 bioclimatic TASs evaluated via comparisons between *G. soja* and landraces.
**Table S11** Allelic status and Fst in 17 flowering time and 27 bioclimatic TASs evaluated via comparisons between pairs of three landrace sub‐populations.

**Table S1** Geographical distribution pattern of 2035 soybean accessions.

**Table S2** Quality trimming of tGBS Reads.
