# Supplementary material for: Identification of loci controlling adaptation in Chinese soya bean landraces via a combination of conventional and bioclimatic GWAS
Source: Plant Biotechnol J. 2019 Jul 24;18(2):389–401. doi: 10.1111/pbi.13206 (PMC6953199; doi:10.1111/pbi.13206)
Supplement: Supplementary file 1 — Figure S1 Summary of 99 085 imputed SNPs. Figure S2 ΔK values as a function of K, the number of putative sub‐clusters within 2035 soybean accessions. Figure S3 Neighbor‐joining tree of 2035 soybean accessions based on shared allele pairwise distances. Figure S4 Pearson's co‐efficient for pairwise comparison of eight variables, including flowering time in Beijing and Wuhan, three geographical, and three climate variables in soybean landraces. Figure S5 Variation in flowering time across three defined sub‐populations of soybean landrace at Beijing and Wuhan locations. Figure S6 Quantile‐quantile plots from GWAS analyses for flowering time data collected from five locations, including Beijing (A), Wuhan (B), Beijing CP (C), Beijing SY (D) and Nanjing (E). Figure S7 Genotype effects (measured as the difference in days between two homozygous genotypes) of the 17 flowering TASs in three sub‐populations of landraces at the Beijing and Wuhan locations. Figure S8 Boxplots for flowering times in three sowing types of SR sub‐population based on the different genotypes of Chr06:19873100 in Beijing and Wuhan locations. Figure S9 Manhattan and quantile‐quantile plots resulting from genome‐wide association studies for bioclimatic variables in soybean landraces. Grey horizontal dashed lines indicate 1% Bonferroni‐corrected genome‐wide significance thresholds, 1.0E‐07. Figure S10 Genotype frequencies in three sub‐populations of landraces inferred from STRUCTURE analysis and the genetic effects on associated flowering times across two locations, Beijing and Wuhan, at nine bioclimatic TASs that are sub‐population‐specific (or with particularly low MAF (<5%) in one and/or two populations). Figure S11 Geographic distributions of genotypes in 1938 Chinese landraces of 17 SNPs associated with flowering time at Beijing and/or Wuhan locations. Blue dots indicate minor homozygous, yellow dots indicate major homozygous and grey dots indicate heterozygous genotypes. Figure S12 Geographic distr [file PBI-18-389-s003.docx]

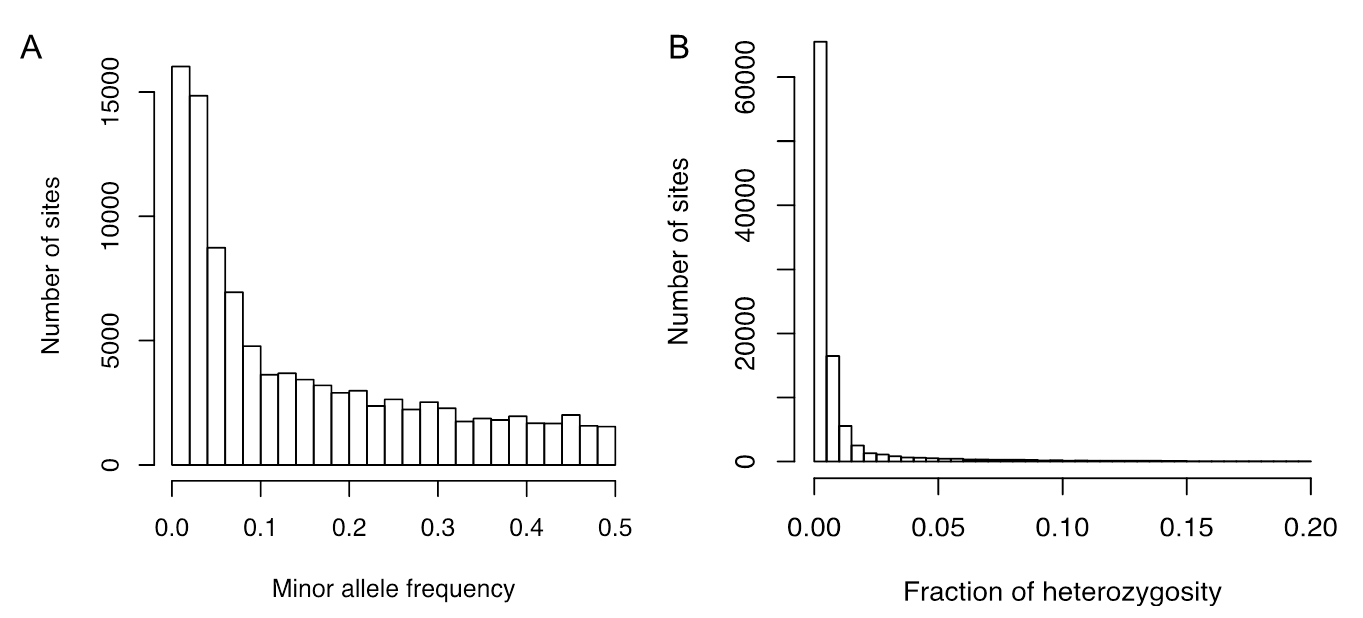


Supplementary Fig. 1 Summary of 99,085 imputed SNPs. (A) Distribution of minor allele frequency. (B) Distribution of fraction of heterozygosity.


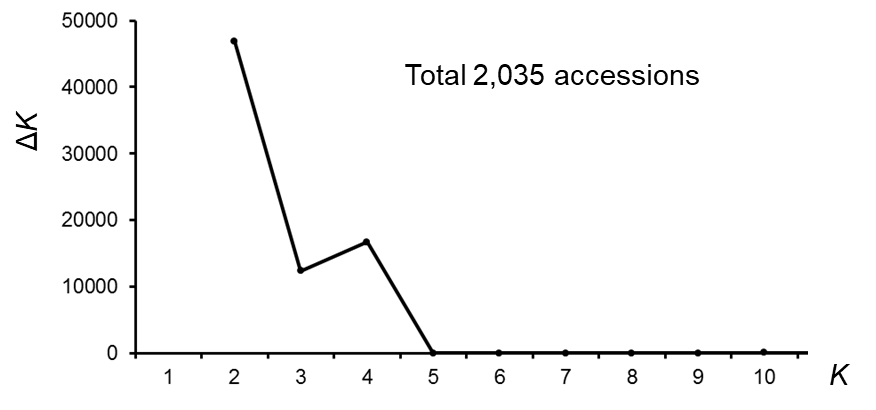


Supplementary Fig. 2 Δ*K* values as a function of *K*, the number of putative sub-clusters within 2,035 soybean accessions.


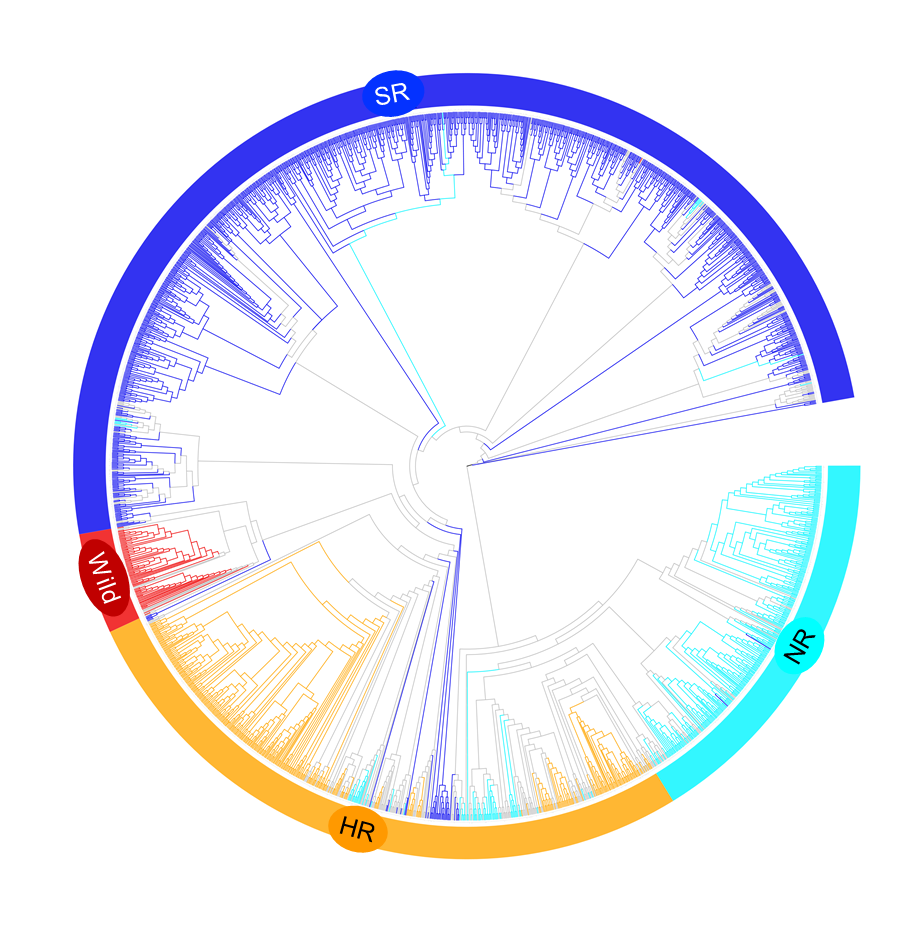


Supplementary Fig. 3 Neighbor-joining tree of 2,035 soybean accessions based on shared allele pairwise distances. Colored lines represented the four defined populations and mixed accessions with admixture genome (Supplementary Table 3).


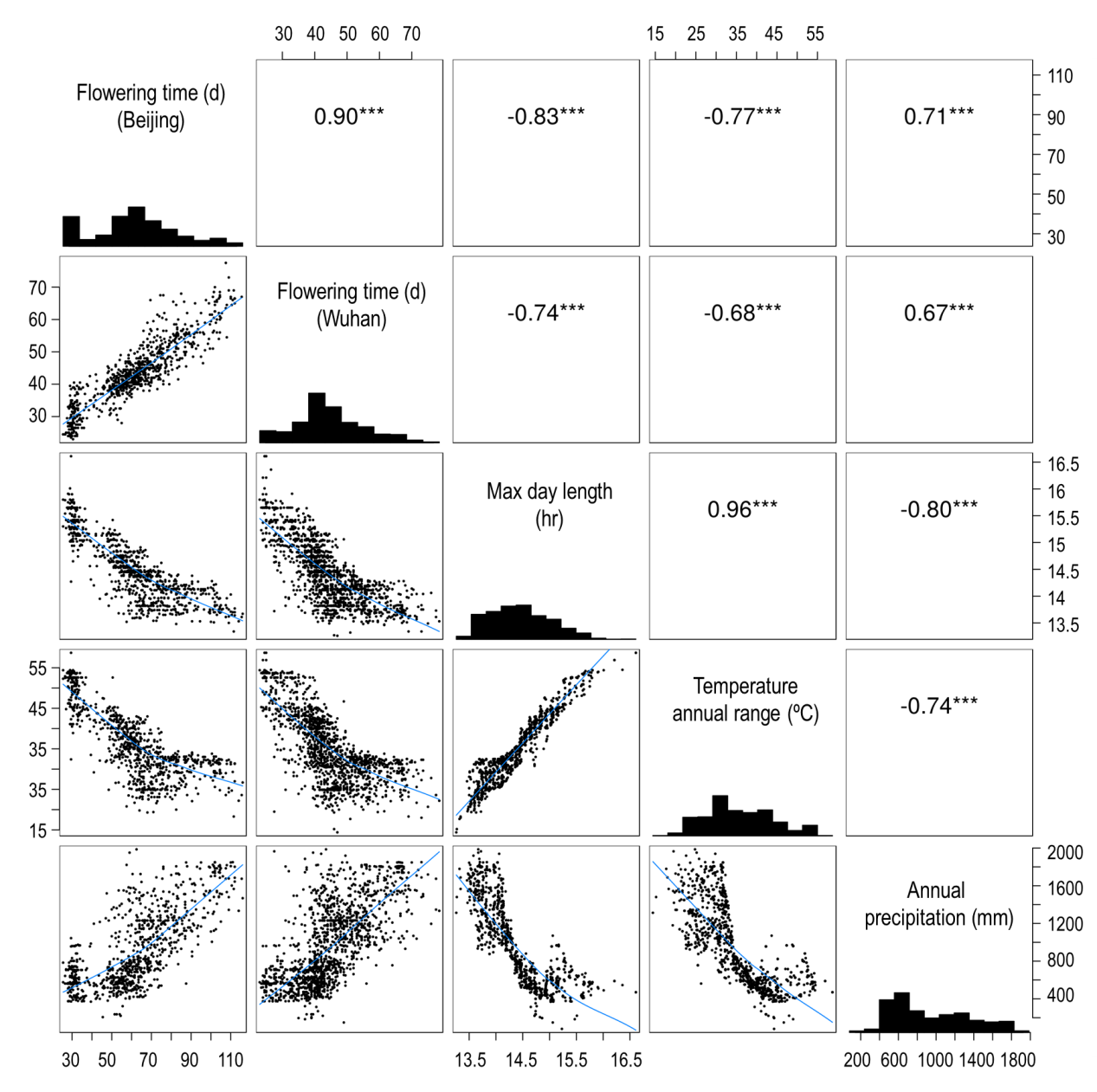


Supplementary Fig. 4 Pearson’s co-efficient for pairwise comparison of five variables, including flowering time in Beijing and Wuhan, and three climate variables in soybean landraces. The "***" shows the significant level of the corresponding two-tail t-test *p* value is 0.001

Supplementary Fig. 5 Variation in flowering time across three defined sub-populations of soybean landrace at Beijing and Wuhan locations. ***, significant at the 0.001 level.


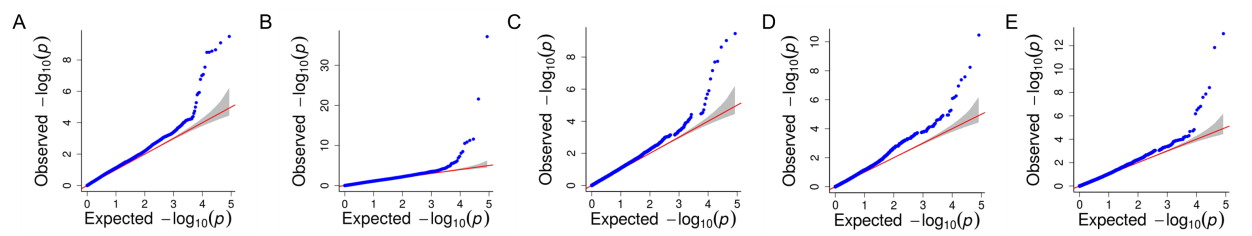


Supplementary Fig. 6 Quantile-quantile plots from GWAS analyses for flowering time data collected from five locations, including Beijing (A), Wuhan (B), BeijingCP (C), BeijingSY (D) and Nanjing (E). Beijing and Wuhan plots are based on the all-landrace panel; the other three plots are based on the core-landrace panel.


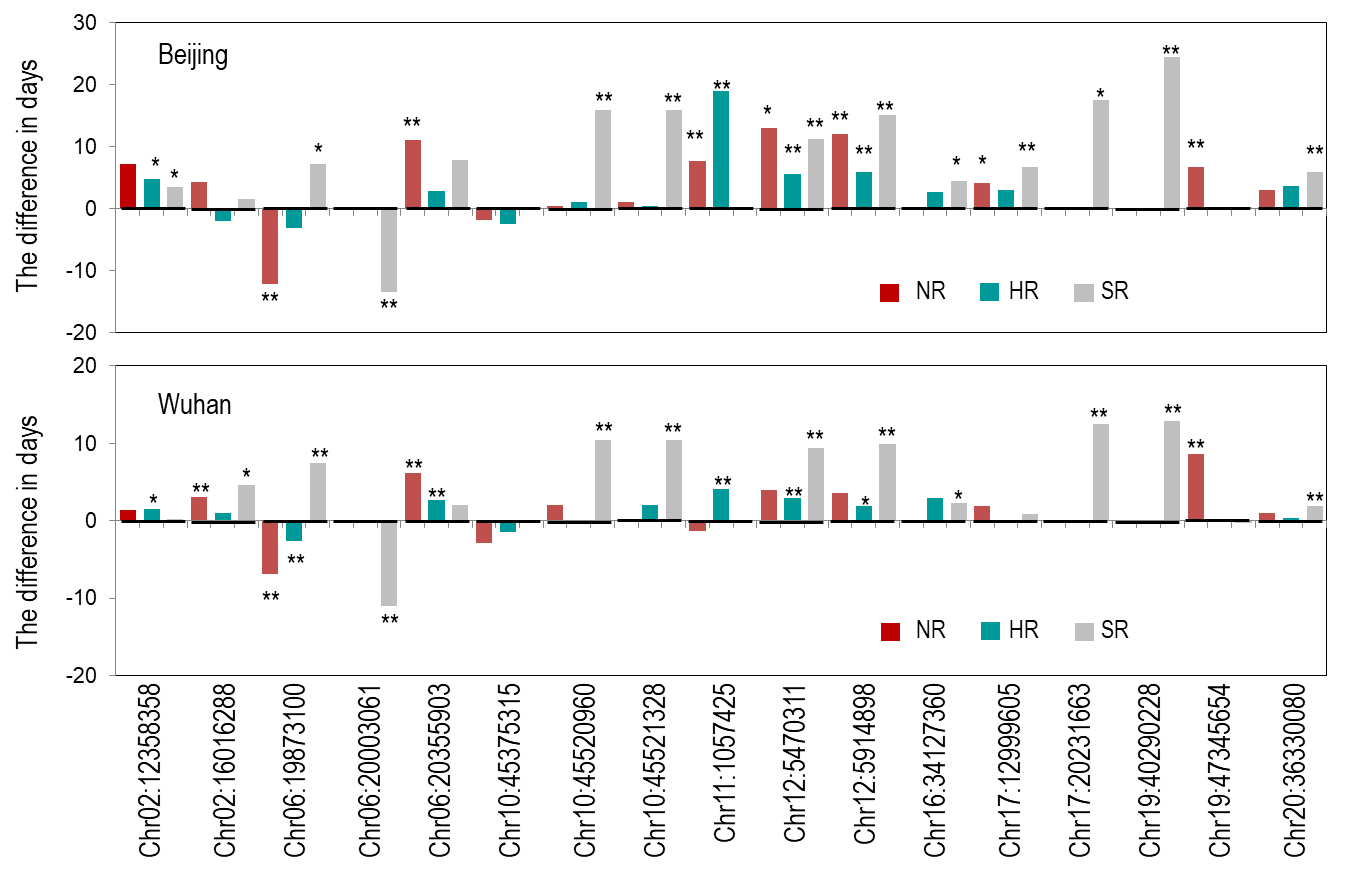


Supplementary Fig. 7 Genotype effects (measured as the difference in days between two homozygous genotypes) of the 17 flowering TASs in three sub-populations of landraces at the Beijing and Wuhan locations.


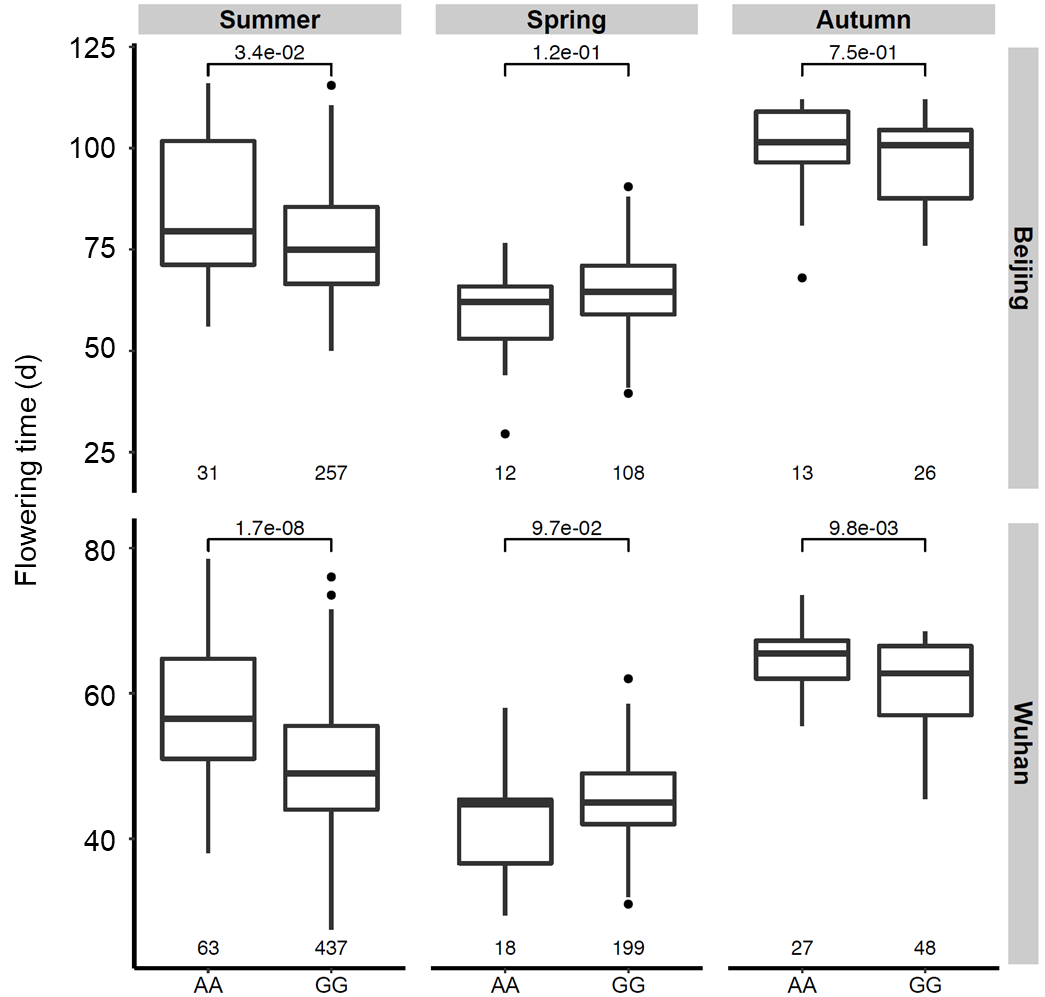


Supplementary Fig. 8 Boxplots for flowering time in three sowing types of SR sub-population based on the different genotypes of Chr06:19873100 in Beijing and Wuhan locations.


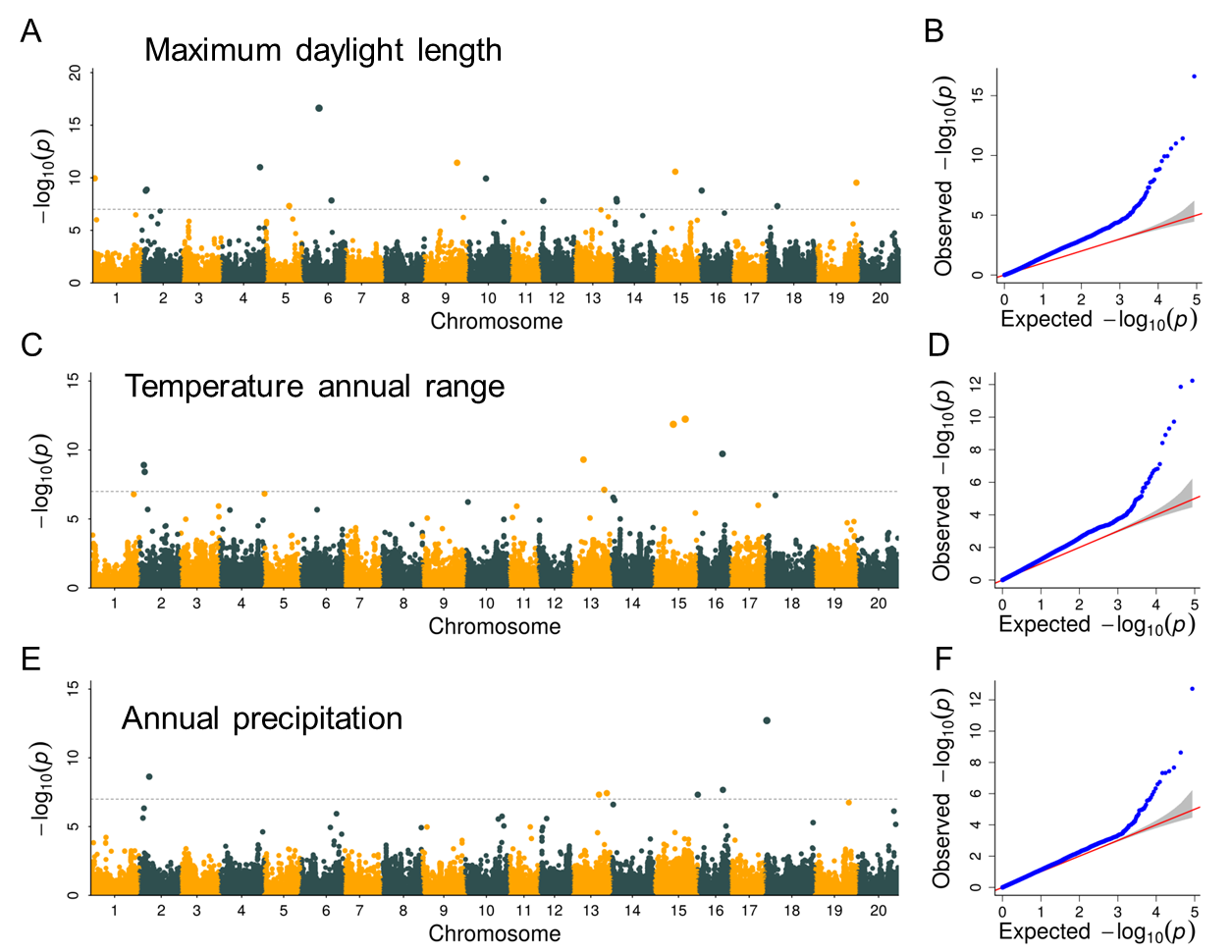


Supplementary Fig. 9 Manhattan and quantile-quantile plots resulting from genome-wide association studies for bioclimatic variables in soybean landraces. (A, B) Temperature annual range. (C, D) Maximum daylight length. (E, F) Annual precipitation. Gray horizontal dashed lines indicate 1% Bonferroni-corrected genome-wide significance thresholds, 1.0E-07.


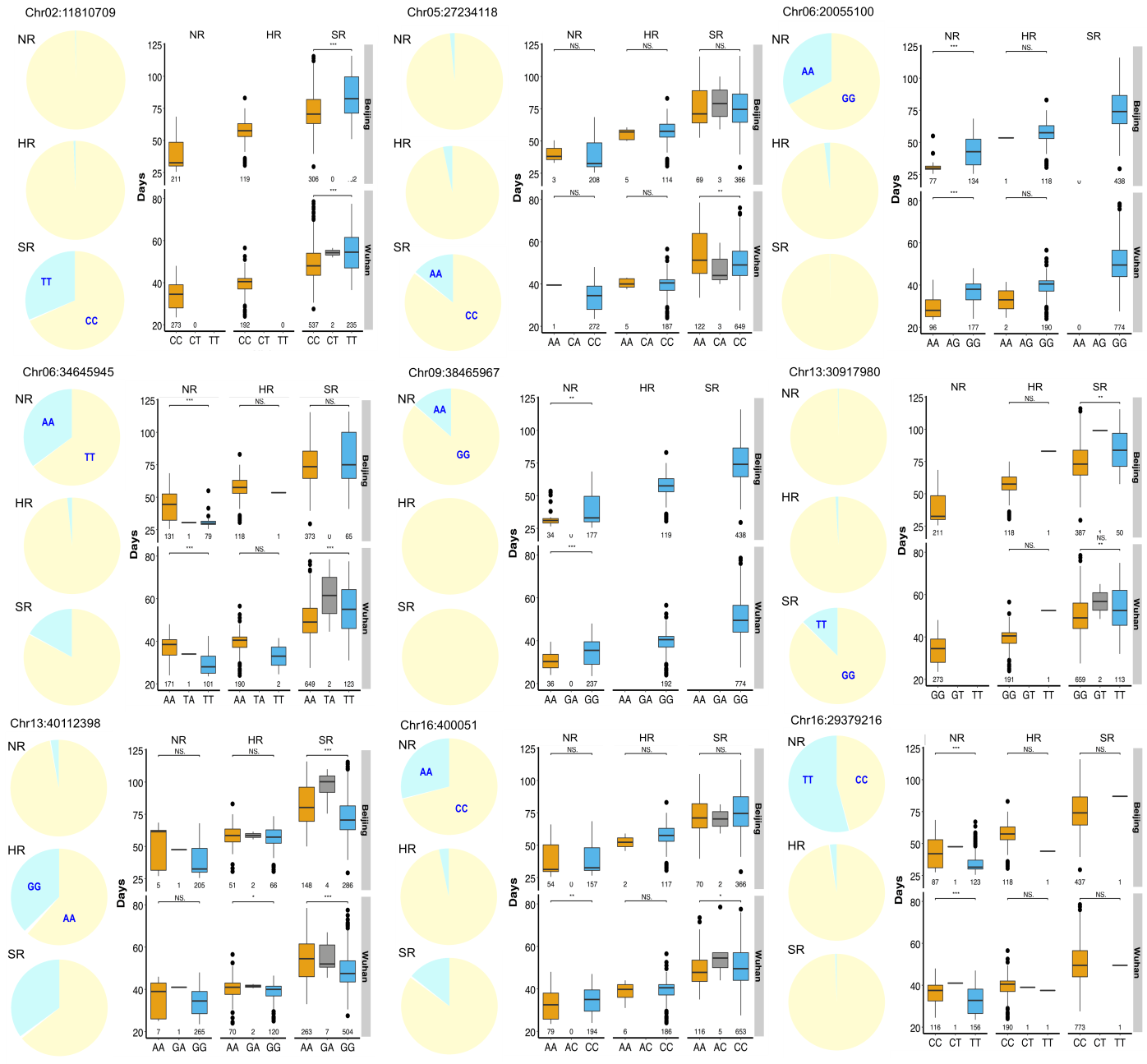


Supplementary Fig. 10 Genotype frequencies in three sub-populations of landraces inferred from STRUCTURE analysis and the genetic effects on associated flowering times across two locations, Beijing and Wuhan, at nine bioclimatic TASs that are sub-population-specific (or with particularly low MAF (<5%) in one and/or two populations).


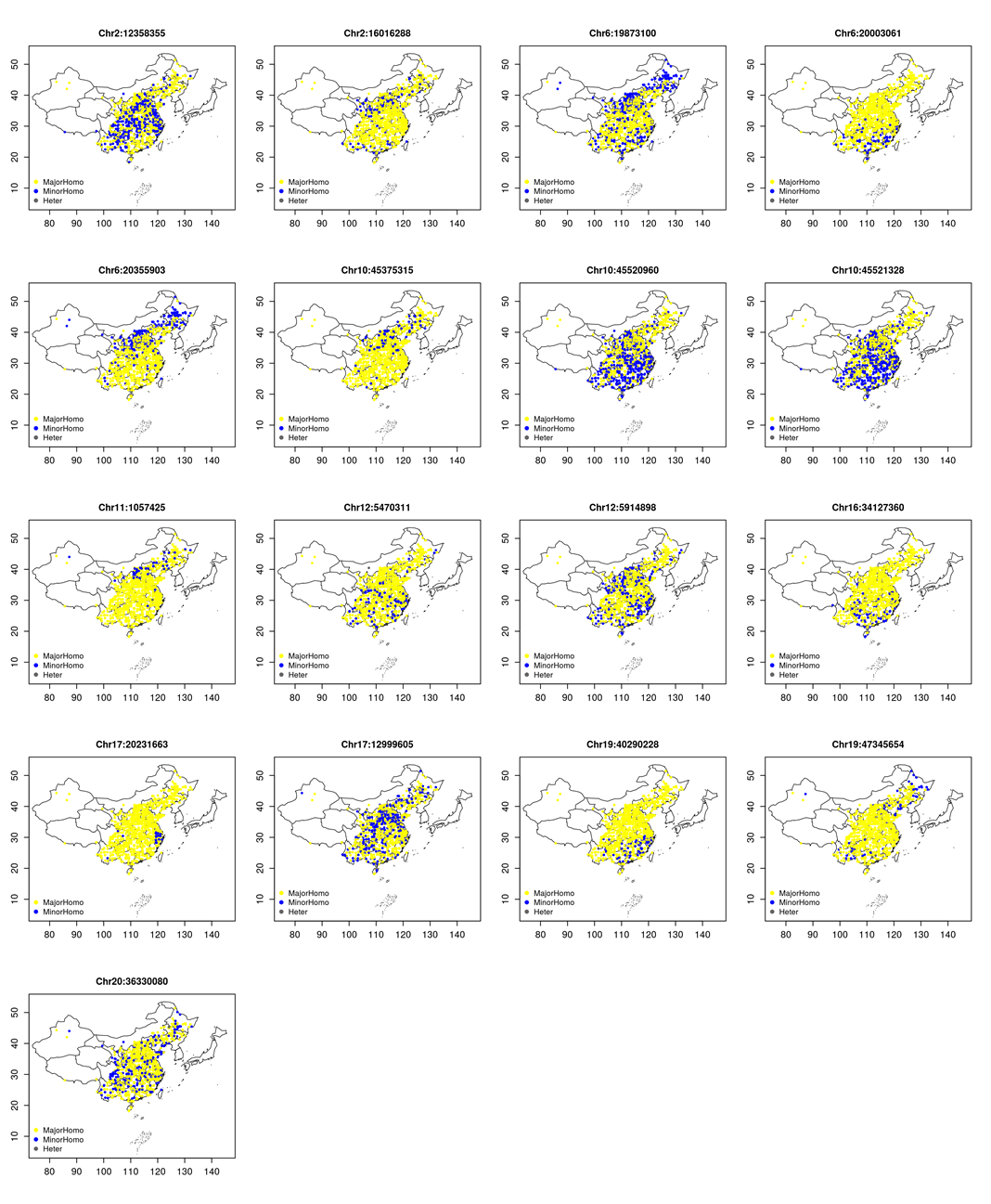


Supplementary Fig. 11 Geographic distributions of genotypes in 1,938 Chinese landraces of 17 SNPs associated with flowering time at Beijing and/or Wuhan locations. Blue dots indicate minor homozygous, yellow dots indicate major homozygous and grey dots indicate heterozygous genotypes.


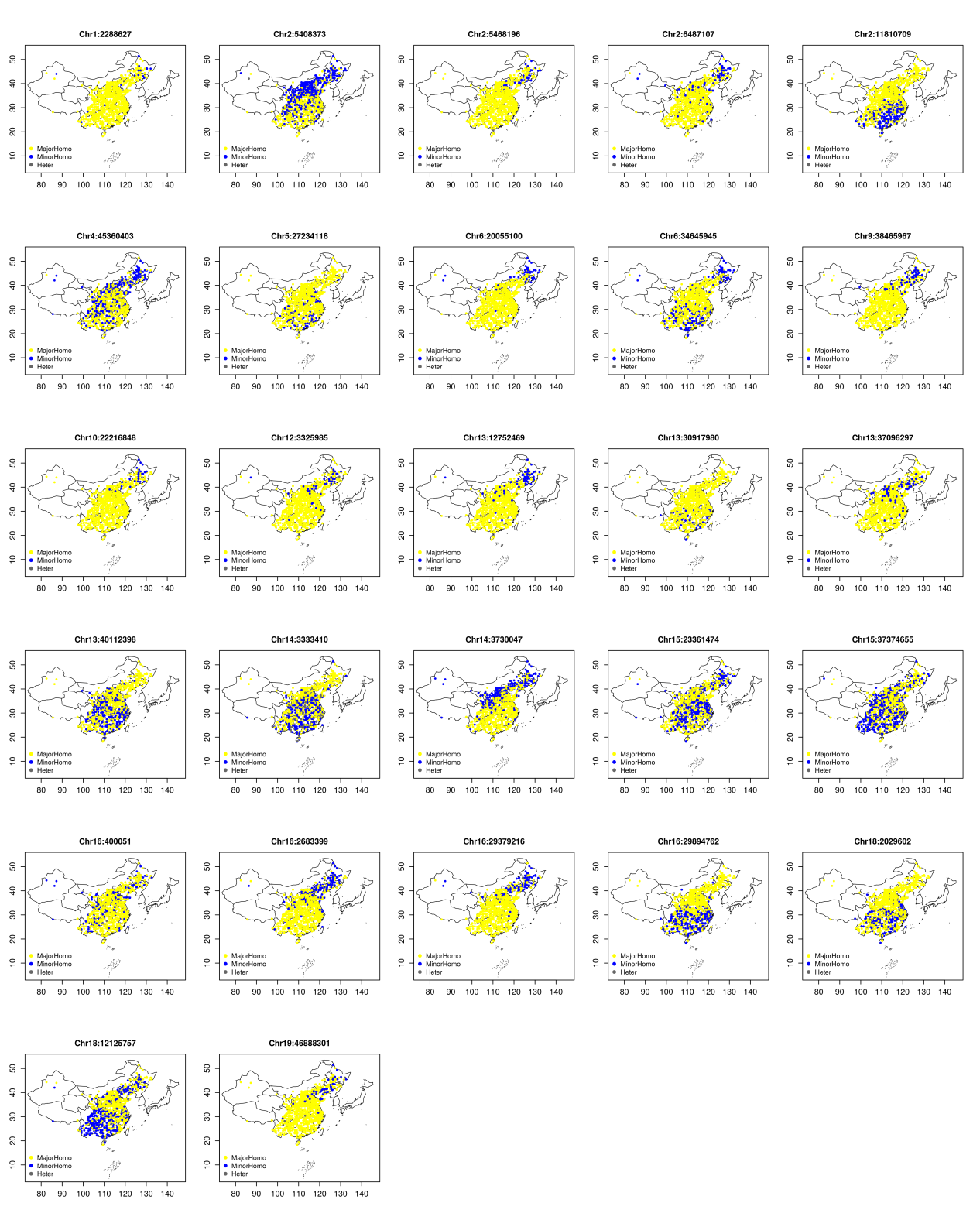


Supplementary Fig. 12 Geographic distributions of genotypes in 27 SNPs associated with bioclimatic variables. Blue dots indicate minor homozygous, yellow dots indicate major homozygous and grey dots indicate heterozygous genotypes.


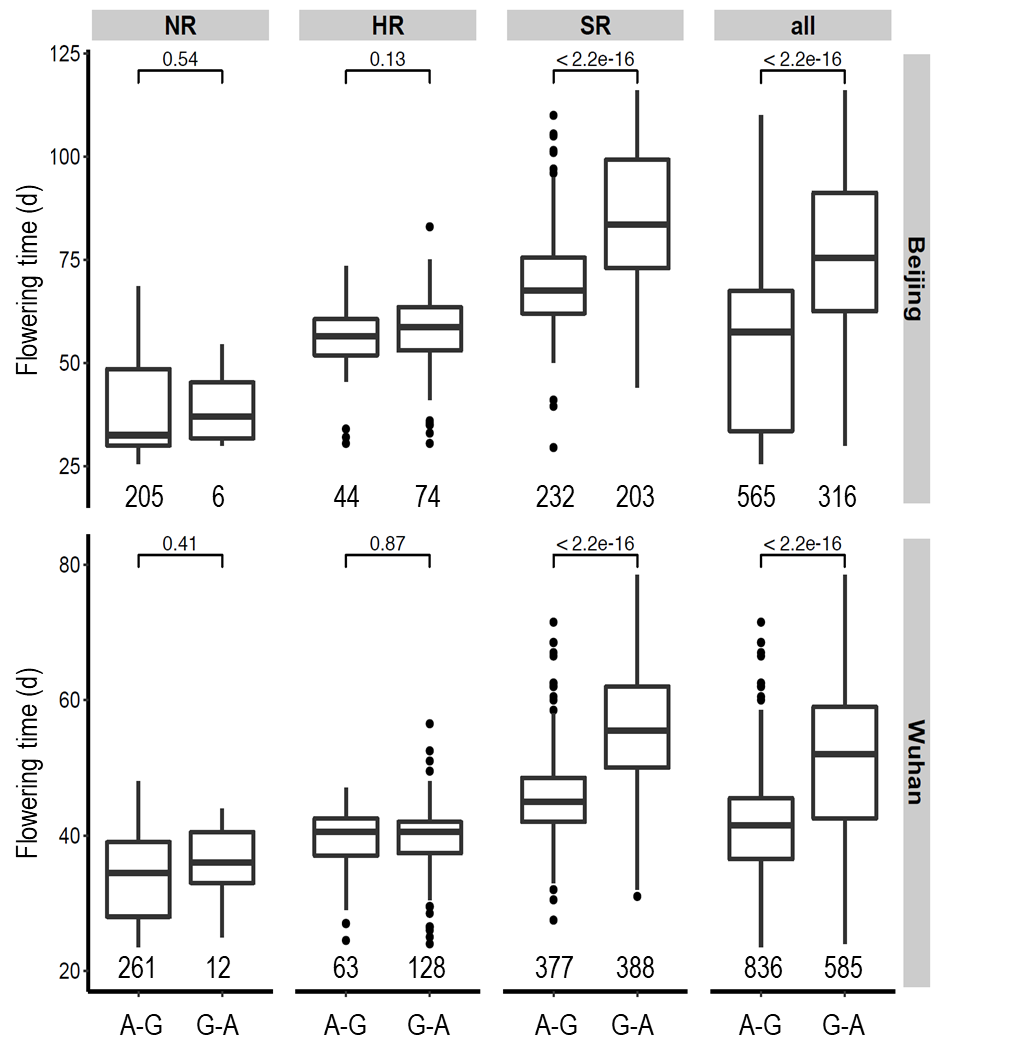


Supplementary Fig. 13 Boxplots for flowering times in three defined sub-populations of landraces based on the two predominately genotypic combinations of Chr10:45520960 and Chr10:45521328. A-G indicated Chr10:45520960-AA_ Chr10:45520960-GG and Chr10:45520960-GG_ Chr10:45520960-AA


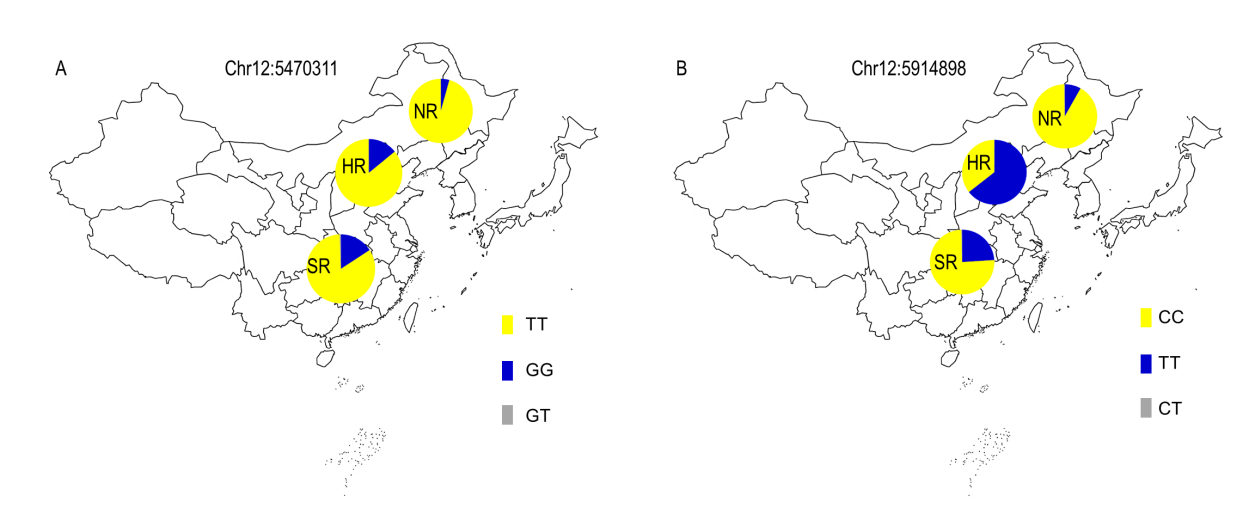


Supplementary Fig. 14 Distribution of genotypes of Chr12:5470311 and Chr12:5914898 in three Chinese sub-populations of landraces. (A) Chr12:5470311. (B) Chr12:5914898.

Supplementary Table 3 No. of soybean landraces in pre-defined species or cultivated ecotypes assigned into populations inferred from STRUCTURE analysis.

| Species | Ecotype* | No. | Populations inferred from STRUCTURE | | | | |
| --- | --- | --- | --- | --- | --- | --- | --- |
|  |  |  | Wild | SR | NR | HR | Mixed |
| *G. soja* | | 97 | 80 | 0 | 0 | 5 | 12 |
| Landrace | NESp | 269 | 0 | 0 | 228 | 29 | 12 |
|  | NSp | 362 | 0 | 7 | 86 | 199 | 70 |
|  | HSp | 26 | 0 | 17 | 1 | 1 | 7 |
|  | HSu | 415 | 0 | 171 | 55 | 109 | 80 |
|  | SSp | 249 | 0 | 223 | 8 | 1 | 17 |
|  | SSu | 529 | 0 | 505 | 7 | 1 | 16 |
|  | SAu | 88 | 0 | 84 | 0 | 1 | 3 |
|  | Sub-total | 1,938 | 0 | 1007 | 385 | 341 | 205 |
| Total |  | 2,035 | 80 | 1007 | 385 | 346 | 217 |

* NESp, Northeast spring-type; NSp, North spring-type; HSp, Huang-Huai spring-type; Hsu, Huang-Huai summer-type; SSp, South spring-type; SSu, South summer-type; SAu, South autumn-type.

Supplementary Table 4 Summary statistics of soybean linkage disequilibrium (*r*^2^) within different sub-groups on accessions.

| Evolutionary types | Defined populations | Sample No. | Decay Length (kb) ^&^ |
| --- | --- | --- | --- |
| ALL |  | 2,035 | 73kb |
| Landrace |  | 1,938 | 145kb |
| *G.soja* |  | 97 | 9kb |
|  | **Wild** | **80** | **9** |
|  | **SR** | **1,007** | **215** |
|  | **NR** | **385** | **385** |
|  | **HR** | **346** | **395** |

^&^, LD was evaluated for SNPs with minor allele frequency (MAF) > 1% and indicated by *r*^2^ dropping to 0.2.

Supplementary Table 5 Summary statistics for genome-wide SNPs and estimates of gene diversity (θw) across 2,035 soybean accessions

| Type1 | Type2* | No. of samples | No. of variants | | | | | | | | | Θw (central 95% range) | Θw (mean) |
| --- | --- | --- | --- | --- | --- | --- | --- | --- | --- | --- | --- | --- | --- |
|  |  |  | Total | Intergenic | Genic | | | | | Upstream | Downstream |  |  |
|  |  |  |  |  | Sub-total | 5′UTR | 3′UTR | Intron | Exon |  |  |  |  |
| Total |  | 2,035 | 99,085 | 53,034 | 13,831 | 832 | 1,638 | 5,434 | 5,927 | 19,623 | 12,597 | 6.90e-06,2.60e-05 | 1.50E-05 |
| *G.soja* |  | 97 | 94,182 | 49,994 | 13,198 | 794 | 1,575 | 5,189 | 5,640 | 18,864 | 12,126 | 7.90e-06,2.94e-05 | 1.69E-05 |
| Landrace |  | 1,938 | 86,818 | 46,025 | 12,364 | 752 | 1,442 | 4,819 | 5,351 | 17,331 | 11,098 | 4.30e-06,2.32e-05 | 1.23E-05 |
|  | Wild | 80 | 92,080 | 48,838 | 12,906 | 781 | 1,549 | 5,086 | 5,490 | 18,471 | 11,865 | 8.00e-06,2.97e-05 | 1.71E-05 |
|  | NR | 385 | 70,860 | 36,392 | 10,423 | 632 | 1,217 | 4,060 | 4,514 | 14,730 | 9,315 | 3.00e-06,2.05e-05 | 1.08E-05 |
|  | HR | 346 | 74,840 | 38,843 | 10,906 | 651 | 1,298 | 4,249 | 4,708 | 15,341 | 9,750 | 4.00e-06,2.20e-05 | 1.16E-05 |
|  | SR | 1,007 | 76,758 | 40,549 | 10,983 | 659 | 1,275 | 4,264 | 4,785 | 15,402 | 9,824 | 3.40e-06,2.23e-05 | 1.16E-05 |

* Four distinct populations inferred from population structure analyses.

Supplementary Table 6 Population differentiations between *G. soja* and landraces and between pairs of four defined sub-populations of landraces

| Comparisons (s1 vs. s2) | No. of Shared SNPs | No. of SNPs specific to s1 | No. of SNPs specific to s2 | Fst |
| --- | --- | --- | --- | --- |
| *G.soja* vs. landraces | 81,915 | 12,267 | 4,903 | 0.27 |
| Wild vs. NR | 66,599 | 25,481 | 4,261 | 0.27 |
| Wild vs. HR | 71,314 | 20,766 | 3,526 | 0.25 |
| Wild vs. SR | 71,328 | 20,752 | 5,430 | 0.31 |
| NR vs. HR | 63,502 | 7,358 | 11,338 | 0.15 |
| NR vs. SR | 62,795 | 8,065 | 13,963 | 0.08 |
| HR vs. SR | 62,954 | 11,886 | 13,804 | 0.18 |

Supplementary Table 7 Evaluation of Pst by comparing mean Qst and Fst estimates for flowering times between pairs of sub-populations of landraces

| Location | Pst (Qst-Fst, c/h^2^ *=1) | | |  | Pst (Qst-Fst, c/h^2^ *=0.6) | | |
| --- | --- | --- | --- | --- | --- | --- | --- |
|  | NR_HR | NR_SR | HR_SR |  | NR_HR | NR_SR | HR_SR |
| Beijing | 0.987 | 0.998 | 0.991 |  | 0.979 | 0.996 | 0.984 |
| Wuhan | 0.963 | 0.997 | 0.994 |  | 0.940 | 0.996 | 0.991 |

* c is the assumed additive genetic proportion of differences between populations, where h^2^ is (narrow-sense heritability) the assumed additive genetic proportion of differences between individuals within populations[^1^](#_ENREF_1)

Supplementary Table 8 17 SNPs associated with flowering time in landraces measured at Beijing and Wuhan locations

| TASs^*^ | *p* value (PVE, %)^ | | LD block surrounding TAS defined by r2=0.8 | | Previously reported QTL ^#^ covered the TAS | Previously reported TAS^#^ located in the LD block | Candidate flowering gene located in the LD block | | |
| --- | --- | --- | --- | --- | --- | --- | --- | --- | --- |
|  | Wuhan (1,433) | Beijing (886) |  |  |  |  | Gene ID | Distance to TAS | Annotation |
| Chr02:12358355 |  | 2.9E-08 (1.1) | 12358318_12358362 | | First flower 16-2 | Chr02_12316110  Chr02_12408115  Chr02_12408988  ss715581063 |  |  |  |
| Chr02:16016288 | 5.3E-08(0.0) |  | 15986767_16591439 | | First flower 13-2 |  |  |  |  |
| Chr06:19873100 | 1.4E-11 (15.0) |  | 19733703_20765160 | | First flower 5-1,12-2, 26-15, 26-17, Vegetative period 1-3 | Chr06_20057204  Chr06_20097064  Chr06_20297543 | *Glyma.06g207800* | 334 | B3 domain containing protein (*GmE1*) [^2^](#_ENREF_2) |
| Chr06:20003061 | 3.0E-11 (8.4) |  | 19926631_20798059 | | First flower 5-1,12-2, 26-15, 26-17, Vegetative period 1-3 | Chr06_20057204  Chr06_20097064  Chr06_20297543 | *Glyma.06g207800* | 204 | B3 domain containing protein (*GmE1*) [^2^](#_ENREF_2) |
| Chr06:20355903 |  | 3.2E-09 (12.4) | 20033051_21233740 | | First flower 8-1, 9-1, 10-1, 20-1, Vegetative period 1-1 |  | *Glyma.06g207800* | 148 | B3 domain containing protein (*GmE1*) [^2^](#_ENREF_2) |
|  |  |  |  | |  |  | *Glyma.06G210600* | 511.6 | TCP family (AtBRC1)[^3^](#_ENREF_3) |
| Chr10:45375315 | 8.4E-09 (0.8) |  | 45313080_45404476 | | First flower 24-4 | Chr10_45310798 | *Glyma.10G222800* | 12.1 | SET domain (*SDG26/ASHH1*) [^4^](#_ENREF_4) |
|  |  |  |  | |  |  | *Glyma.10G221500* | 59.2 | Gigantea (*GmE2*) [^5^](#_ENREF_5) |
| Chr10:45520960 | 7.0E-38 (16.5) |  | 45426978_45521407 | | First flower 24-4 | Chr10_45465189 |  |  |  |
| Chr10:45521328 |  | 8.1E-10 (12.0) | 45520960_45521407 | | First flower 24-4 |  |  |  |  |
| Chr11:1057425 |  | 3.3E-09 (0.3) | 1038304_1176434 | |  | ss715608856 |  |  |  |
| Chr12:5470311 | 2.6E-22 (0.9) |  | 5470301_ 5563152 | | First flower 25-2 | Chr12_5475503 | *Glyma.12G073900* | 38.1 | pseudo-response regulator (*AtPRR7*)[^6^](#_ENREF_6) |
|  |  |  |  |  |  | Chr12_5504773 | *Glyma.12G073300* | 40.1 | AP2 domain (*AtTOE1*)[^7^](#_ENREF_7) |
| Chr12:5914898 | 5.4E-12 (1.8) | 2.7E-09 (2.3) | 5903229_5976730 | | First flower 25-2 | ss715613207 |  |  |  |
| Chr16:34127360 | 3.3E-09 (2.5) |  | 34106577_34148988 | |  |  |  |  |  |
| Chr17:12999605 |  | 8.3E-08 (0.0) | 12932243_12999635 | |  |  |  |  |  |
| Chr17:20231663 | 2.6E-12 (0.0) |  | 19419004_21119007 | |  |  |  |  |  |
| Chr19:40290228 |  | 2.2E-09 (4.0) | 40290216_40290303 | | First flower 15-2 |  |  |  |  |
| Chr19:47345654 |  | 9.9E-08 (0.8) | 47334168_47349893 | | First flower 13-9, 16-4, 20-2 | Satt229 |  |  |  |
| Chr20:36330080 |  | 3.2E-10 (0.0) | 36321749_36331089 | | First flower 25-3 |  |  |  |  |

^*^ Chr and the following two digit number in front of colon represents the chromosome number, and the number after the colon represents the physical location of TAS. ^#^ The flowering time-related QTL and TAS reported on Soybase (http://www.soybase.org). ^ PVE, phenotypic variation explained.

Supplementary Table 9 27 TASs associated with three bioclimatic variables (temperature annual range (TAR), annual precipitation (AP) and maximum daylight length (MDL)).

| TASs^*^ | *p* value | | | LD block surrounding TAS defined by r2=0.8 | Previously reported QTL ^#^ covered the TAS | Previously reported TAS^#^ located in the LD block | Candidate flowering gene located in the LD block | | |
| --- | --- | --- | --- | --- | --- | --- | --- | --- | --- |
|  | MDL | TAR | AP |  |  |  | Gene ID | Distance to TAS (kb) | Annotation |
| Chr01:2288627 | 1.1E-10 |  |  | 2108437- 2289734 |  |  |  |  |  |
| Chr02:5408373 | 1.7E-09 |  |  | 4417798_6309030 |  |  | Glyma.02G060500 | 64.9 | Set domain (*AtSUVR5*)[^8^](#_ENREF_8) |
| Chr02:5468196 |  | 1.2E-09 |  | 4515828_5488224 |  |  | Glyma.02G060500 | 5.1 | Set domain (*AtSUVR5*)[^8^](#_ENREF_8) |
| Chr02:6487107 | 1.3E-09 | 3.9E-09 |  | 6047164_6526363 |  |  | Glyma.02G069200 | 386.4 | PEBP (Gm*FT2c*)[^9^](#_ENREF_9) |
|  |  |  |  |  |  |  | Glyma.02G069500 | 369.7 | PEBP (Gm*FT2d*)[^9^](#_ENREF_9) |
| Chr02:11810709 |  |  | 2.3E-09 | 10926263_12793005 | First flower 16-2 | Chr02_12316110, Chr02_12408115, Chr02_12408988 | Glyma.02G121600 | 274 | MADS-box (*AtAGL7*, *AP1*)[^10^](#_ENREF_10) |
| Chr04:45360403 | 9.9E-12 |  |  | 44400037_45394435 |  |  |  |  |  |
| Chr05:27234118 | 4.8E-08 |  |  | 27032090_27810712 |  |  |  |  |  |
| Chr06:20055100 | 2.4E-17 |  |  | 19280668_21052310 | First flower 4-1, 26-9, 26-12 | Chr06_20057204, Chr06_20097064, Chr06_20297543, Chr06_20299454 | Glyma.06G207800 | 152 | B3 domain containing protein (*GmE1*)[^2^](#_ENREF_2) |
|  |  |  |  |  |  |  | Glyma.06G205800 | 464.2 | AGAMOUS-like family (*GmFULa*)[^11^](#_ENREF_11) |
|  |  |  |  |  |  |  | Glyma.06G210600 | 812.4 | TCP family (*AtBRC1*)[^3^](#_ENREF_3) |
| Chr06:34645945 | 1.4E-08 |  |  | 33681396_35641511 | First flower 3-1 |  |  |  |  |
| Chr09:38465967 | 3.7E-12 |  |  | 37878641_38958636 |  |  | Glyma.09G158400 | 284.2 | Nuclear factor Y(*AtHAP3a*)[^12^](#_ENREF_12) |
|  |  |  |  |  |  |  | Glyma.09G156500 | 479.5 | Histone-lysine N-methyltransferase (*AtSDG7*)[^13^](#_ENREF_13) |
| Chr10:22216848 | 1.2E-10 |  |  | 22216817_22216875 |  |  |  |  |  |
| Chr12:3325985 | 1.6E-08 |  |  | 3204652_4034328 |  |  | Glyma.12G048600 | 178.9 | AP2/B3-like family (*AtVRN1*) [^14^](#_ENREF_14) |
|  |  |  |  |  |  |  | Glyma.12G051000 | 338.7 | RNA binding (*AtFPA*)[^15^](#_ENREF_15) |
| Chr13:12752469 |  | 5.0E-10 |  | 12184320_13240787 | First flower 25-1, 11-4 |  |  |  |  |
| Chr13:30917980 |  |  | 4.7E-08 | 29922363_31858503 |  |  |  |  |  |
| Chr13:37096297 |  | 7.7E-08 |  | 37087597_37330936 |  |  |  |  |  |
| Chr13:40112398 |  |  | 3.7E-08 | 39587005_40536613 |  |  |  |  |  |
| Chr14:3333410 | 1.1E-08 |  |  | 3330965_3333445 |  |  |  |  |  |
| Chr14:3730047 | 1.8E-08 |  |  | 3335439_3803461 |  |  | Glyma.14G044500 | 349.1 | Ubiquitin-specific protease (*AtUBP12*)[^16^](#_ENREF_16) |
| Chr15:23361474 | 2.7E-11 | 1.4E-12 |  | 22369866_24322139 |  |  | Glyma.15G196500 | 671.5 | Phytochrome E (*AtPHYE*)[^17^](#_ENREF_17) |
| Chr15:37374655 |  | 5.8E-13 |  | 36387580_38325063 |  |  |  |  |  |
| Chr16:400051 |  |  | 4.8E-08 | 56602_442349 |  |  |  |  |  |
| Chr16:2683399 | 1.6E-09 |  |  | 2664597_3667696 |  |  | Glyma.16G029600 | 126 | POZ/BTB containin G-protein (*AtLRB2*)[^18^](#_ENREF_18) |
|  |  |  |  |  |  |  | Glyma.16G035100 | 637.4 | Transcription regulatory protein (*AtBRM*)[^19^](#_ENREF_19) |
| Chr16:29379216 |  | 1.9E-10 |  | 28861842_30221405 | First flower 9-3 |  |  |  |  |
| Chr16:29894762 |  |  | 2.1E-08 | 29154142_30187284 | First flower 9-3 |  |  |  |  |
| Chr18:2029602 |  |  | 1.9E-13 | 1431186_2194706 |  |  | Glyma.18G027500 | 34 | Protein of unknown function (*AtELF4, GmELF4*) [^20^](#_ENREF_20)^,^[^21^](#_ENREF_21) |
| Chr18:12125757 | 4.9E-08 |  |  | 11731088_13117704 |  |  |  |  |  |
| Chr19:46888301 | 2.9E-10 |  |  | 46502353_46973004 | First flower 13-9, 16-4, 20-2 |  | Glyma.19G216700 | 81.3 | RGA-like (*AtRGL1*) [^22^](#_ENREF_22) |

^*^ Chr and the following two-digit number in front of colon represents the chromosome number, and the number after the colon represents the physical location of TAS. ^#^ The flowering time-related QTL and TAS reported on Soybase (http://www.soybase.org).

Supplementary Table 10 Allelic status and Fst in 17 flowering time and 27 bioclimatic TASs evaluated via comparisons between *G. soja* and landraces

| TAS* | Trait | Polymorphism^ | Frequency of Reference allele | | Fst (*G. soja* vs. Landrace) |
| --- | --- | --- | --- | --- | --- |
|  |  |  | *G. soja* (97 accessions) | Landrace (1938 accessions) |  |
| Chr01:2288627 | MDL | C/T | 0.959 | 0.991 | 0.043 |
| Chr02:5408373* | MDL | A/G | 0.160 | 0.520 | 0.207 |
| Chr02:5468196 | TAR | A/C | 0.995 | 0.941 | 0.021 |
| Chr02:6487107 | MDL, TAR | A/G | 0.031 | 0.040 | 0.000 |
| Chr02:11810709 | AP | C/T | 0.969 | 0.833 | 0.060 |
| Chr02:12358355* | Flowering time | C/T | 0.814 | 0.396 | 0.268 |
| Chr02:16016288 | Flowering time | C/T | 0.763 | 0.870 | 0.043 |
| Chr04:45360403 | MDL | T/C | 0.423 | 0.654 | 0.101 |
| Chr05:27234118 | MDL | C/A | 0.701 | 0.912 | 0.200 |
| Chr06:19873100* | Flowering time | A/G | 0.830 | 0.343 | 0.346 |
| Chr06:20003061* | Flowering time | T/C | 0.629 | 0.941 | 0.432 |
| Chr06:20055100 | MDL | A/G | 0.186 | 0.076 | 0.071 |
| Chr06:20355903 | Flowering time | C/A | 0.175 | 0.275 | 0.019 |
| Chr06:34645945* | MDL | T/A | 0.521 | 0.173 | 0.287 |
| Chr09:38465967 | MDL | G/A | 1.000 | 0.972 | 0.010 |
| Chr10:22216848 | MDL | C/T | 1.000 | 0.980 | 0.005 |
| Chr10:45375315* | Flowering time | T/G | 0.691 | 0.966 | 0.475 |
| Chr10:45520960 | Flowering time | G/A | 0.253 | 0.423 | 0.052 |
| Chr10:45521328 | Flowering time | A/G | 0.242 | 0.425 | 0.060 |
| Chr11:1057425* | Flowering time | C/T | 0.711 | 0.973 | 0.498 |
| Chr12:3325985 | MDL | T/C | 0.969 | 0.974 | 0.000 |
| Chr12:5470311* | Flowering time | T/G | 0.314 | 0.873 | 0.573 |
| Chr12:5914898* | Flowering time | C/T | 0.108 | 0.729 | 0.498 |
| Chr13:12752469 | TAR | C/T | 0.918 | 0.956 | 0.012 |
| Chr13:30917980 | AP | G/T | 0.990 | 0.933 | 0.021 |
| Chr13:37096297 | TAR | A/T | 0.876 | 0.965 | 0.093 |
| Chr13:40112398 | AP | G/A | 0.521 | 0.724 | 0.088 |
| Chr14:3333410 | MDL | T/A | 0.680 | 0.667 | 0.000 |
| Chr14:3730047 | MDL | C/G | 0.711 | 0.674 | 0.000 |
| Chr15:23361474 | MDL , TAR | G/A | 0.747 | 0.621 | 0.028 |
| Chr15:37374655 | TAR | G/A | 0.423 | 0.591 | 0.051 |
| Chr16:400051 | AP | A/C | 0.093 | 0.170 | 0.016 |
| Chr16:2683399 | MDL | T/G | 0.144 | 0.190 | 0.002 |
| Chr16:29379216* | TAR | C/T | 0.928 | 0.876 | 0.007 |
| Chr16:29894762 | AP | A/G | 0.371 | 0.735 | 0.249 |
| Chr16:34127360 | Flowering time | A/G | 0.778 | 0.915 | 0.097 |
| Chr17:12999605 | Flowering time | T/A | 0.928 | 0.640 | 0.153 |
| Chr17:20231663* | Flowering time | A/C | 0.732 | 0.990 | 0.634 |
| Chr18:2029602 | AP | G/T | 0.887 | 0.824 | 0.008 |
| Chr18:12125757* | MDL | A/T | 0.082 | 0.371 | 0.152 |
| Chr19:40290228 | Flowering time | C/T | 0.985 | 0.941 | 0.012 |
| Chr19:46888301* | MDL | T/A | 0.263 | 0.973 | 0.879 |
| Chr19:47345654* | Flowering time | C/G | 0.356 | 0.904 | 0.615 |
| Chr20:36330080 | Flowering time | G/C | 0.108 | 0.264 | 0.055 |

* The TASs exhibited strong differentiation between wild and landrace accessions.

^ The allele above slash indicated the allele of reference genome Wm82.a2.v1.

Supplementary Table 11 Allelic status and Fst in 17 flowering time and 27 bioclimatic TASs evaluated via comparisons between pairs of three landrace sub-populations

| TAS | Trait | Polymorphism^ | Frequency of Reference allele | | |  | Fst | | | Type* |
| --- | --- | --- | --- | --- | --- | --- | --- | --- | --- | --- |
|  |  |  | NR (385 accessions) | HR (346 accessions) | SR (1007 accessions) |  | NR:HR | NR:SR | HR:SR |  |
| Chr01:2288627 | MDL | C/T | 0.970 | 1.000 | 0.997 |  | 0.026 | 0.033 | 0.000 |  |
| Chr02:11810709 | AP | C/T | 0.999 | 0.994 | 0.691 |  | 0.000 | 0.233 | 0.222 | SR specific |
| Chr02:12358355 | Flowering time | C/T | 0.091 | 0.520 | 0.494 |  | 0.361 | 0.284 | 0.000 | HR and SR specific |
| Chr02:16016288 | Flowering time | C/T | 0.830 | 0.737 | 0.957 |  | 0.023 | 0.103 | 0.230 |  |
| Chr02:5408373 | MDL | A/G | 0.344 | 0.104 | 0.754 |  | 0.148 | 0.298 | 0.566 | Minor allele changed |
| Chr02:5468196 | TAR | A/C | 0.721 | 1.000 | 0.996 |  | 0.267 | 0.390 | 0.001 | NR specific |
| Chr02:6487107 | MDL, TAR | A/G | 0.174 | 0.006 | 0.004 |  | 0.151 | 0.252 | 0.000 | NR specific |
| Chr04:45360403 | MDL | T/C | 0.317 | 0.763 | 0.760 |  | 0.331 | 0.338 | 0.000 | Minor allele changed |
| Chr05:27234118 | MDL | C/A | 0.979 | 0.962 | 0.858 |  | 0.002 | 0.071 | 0.050 | SR specific |
| Chr06:19873100 | Flowering time | A/G | 0.421 | 0.827 | 0.150 |  | 0.294 | 0.185 | 0.635 | Minor allele changed |
| Chr06:20003061 | Flowering time | T/C | 1.000 | 0.997 | 0.893 |  | 0.000 | 0.075 | 0.068 | SR specific |
| Chr06:20055100 | MDL | A/G | 0.325 | 0.035 | 0.001 |  | 0.240 | 0.457 | 0.052 | NR specific |
| Chr06:20355903 | Flowering time | C/A | 0.386 | 0.825 | 0.037 |  | 0.332 | 0.400 | 0.831 | NR and HR specific |
| Chr06:34645945 | MDL | T/A | 0.349 | 0.026 | 0.167 |  | 0.282 | 0.090 | 0.081 | NR and SR specific |
| Chr09:38465967 | MDL | G/A | 0.869 | 0.997 | 1.000 |  | 0.116 | 0.213 | 0.004 | NR specific |
| Chr10:22216848 | MDL | C/T | 0.904 | 1.000 | 1.000 |  | 0.089 | 0.156 | 0.000 | NR specific |
| Chr10:45375315 | Flowering time | T/G | 0.951 | 0.895 | 0.994 |  | 0.020 | 0.050 | 0.146 | HR specific |
| Chr10:45520960 | Flowering time | G/A | 0.056 | 0.669 | 0.510 |  | 0.585 | 0.344 | 0.048 | Minor allele changed |
| Chr10:45521328 | Flowering time | A/G | 0.056 | 0.671 | 0.511 |  | 0.587 | 0.345 | 0.048 | Minor allele changed |
| Chr11:1057425 | Flowering time | C/T | 0.971 | 0.890 | 1.000 |  | 0.049 | 0.045 | 0.189 | HR specific |
| Chr12:3325985 | MDL | T/C | 0.883 | 1.000 | 0.997 |  | 0.109 | 0.173 | 0.000 | NR specific |
| Chr12:5470311 | Flowering time | T/G | 0.952 | 0.858 | 0.841 |  | 0.048 | 0.052 | 0.000 |  |
| Chr12:5914898 | Flowering time | C/T | 0.916 | 0.384 | 0.763 |  | 0.479 | 0.069 | 0.268 | Minor allele changed |
| Chr13:12752469 | TAR | C/T | 0.808 | 0.991 | 1.000 |  | 0.162 | 0.297 | 0.011 | NR specific |
| Chr13:30917980 | AP | G/T | 0.997 | 0.991 | 0.875 |  | 0.001 | 0.084 | 0.073 | SR specific |
| Chr13:37096297 | TAR | A/T | 0.875 | 0.986 | 0.992 |  | 0.084 | 0.158 | 0.000 | NR specific |
| Chr13:40112398 | AP | G/A | 0.962 | 0.630 | 0.651 |  | 0.297 | 0.216 | 0.000 | HR and SR specific |
| Chr14:3333410 | MDL | T/A | 0.894 | 0.756 | 0.540 |  | 0.062 | 0.232 | 0.090 |  |
| Chr14:3730047 | MDL | C/G | 0.404 | 0.418 | 0.873 |  | 0.000 | 0.427 | 0.416 | Minor allele changed |
| Chr15:23361474 | MDL , TAR | G/A | 0.645 | 0.744 | 0.514 |  | 0.020 | 0.032 | 0.099 |  |
| Chr15:37374655 | TAR | G/A | 0.725 | 0.743 | 0.494 |  | 0.000 | 0.100 | 0.115 |  |
| Chr16:2683399 | MDL | T/G | 0.699 | 0.051 | 0.035 |  | 0.610 | 0.727 | 0.001 | NR specific |
| Chr16:29379216 | TAR | C/T | 0.477 | 0.952 | 0.995 |  | 0.424 | 0.647 | 0.052 | NR specific |
| Chr16:29894762 | AP | A/G | 0.970 | 0.987 | 0.531 |  | 0.004 | 0.337 | 0.354 | SR specific |
| Chr16:34127360 | Flowering time | A/G | 1.000 | 0.991 | 0.846 |  | 0.006 | 0.111 | 0.095 | SR specific |
| Chr16:400051 | AP | A/C | 0.282 | 0.038 | 0.150 |  | 0.192 | 0.054 | 0.055 | NR and SR specific |
| Chr17:12999605 | Flowering time | T/A | 0.818 | 0.387 | 0.676 |  | 0.326 | 0.047 | 0.155 | Minor allele changed |
| Chr17:20231663 | Flowering time | A/C | 1.000 | 1.000 | 0.980 |  | NA | 0.012 | 0.012 |  |
| Chr18:12125757 | MDL | A/T | 0.477 | 0.029 | 0.475 |  | 0.408 | 0.000 | 0.339 | NR and SR specific |
| Chr18:2029602 | AP | G/T | 0.990 | 0.965 | 0.692 |  | 0.011 | 0.219 | 0.181 | SR specific |
| Chr19:40290228 | Flowering time | C/T | 1.000 | 0.993 | 0.893 |  | 0.005 | 0.075 | 0.062 | SR specific |
| Chr19:46888301 | MDL | T/A | 0.896 | 0.996 | 0.997 |  | 0.086 | 0.150 | 0.000 | NR specific |
| Chr19:47345654 | Flowering time | C/G | 0.769 | 0.986 | 0.930 |  | 0.187 | 0.117 | 0.027 |  |
| Chr20:36330080 | Flowering time | G/C | 0.282 | 0.111 | 0.304 |  | 0.084 | 0.000 | 0.091 |  |

^ The allele above slash indicated the allele of reference genome Wm82.a2.v1.

* Sub-population specific TASs indicated their minor allele was found only in geographically constrained sets of landraces or exhibited low MAF (<0.05) in one or two sub-populations

.

References

1. Leinonen, T., McCairns, R., O'hara, R. & Merilä, J. Q _ST_-F _ST_ comparisons: evolutionary and ecological insights from genomic heterogeneity. *Nat Reviews Genet* **14**, 179 (2013).

2. Xia, Z. *et al.* Positional cloning and characterization reveal the molecular basis for soybean maturity locus *E1* that regulates photoperiodic flowering. *P Natl Acad Sci USA* **109**, E2155-E2164 (2012).

3. Niwa, M. *et al.* *BRANCHED1* interacts with *FLOWERING LOCUS T* to repress the floral transition of the axillary meristems in *Arabidopsis*. *Plant Cell* **25**, 1228-1242 (2013).

4. Xu, L. *et al.* Di- and tri- but not monomethylation on histone H3 lysine 36 marks active transcription of genes involved in flowering time regulation and other processes in *Arabidopsis thaliana*. *Mol Cell Biol* **28**, 1348-1360 (2008).

5. Watanabe, S. *et al.* A map-based cloning strategy employing a residual heterozygous line reveals that the *GIGANTEA* gene is involved in soybean maturity and flowering. *Genetics* **188**, 395-407 (2011).

6. Yamamoto, Y. *et al.* Comparative genetic studies on the *APRR5* and *APRR7* genes belonging to the APRR1/TOC1 quintet implicated in circadian rhythm, control of flowering time, and early photomorphogenesis. *Plant Cell Physiol* **44**, 1119-1130 (2003).

7. Aukerman, M.J. & Sakai, H. Regulation of flowering time and floral organ identity by a microRNA and its *APETALA2*-like target genes. *Plant Cell* **15**, 2730-2741 (2003).

8. Caro, E. *et al.* The SET-domain protein SUVR5 mediates H3K9me2 deposition and silencing at stimulus response genes in a DNA methylation-independent manner. *PLoS Genet* **8**, e1002995 (2012).

9. Wu, F., Sedivy, E., Price, W., Haider, W. & Hanzawa, Y. Evolutionary trajectories of duplicated FT homologues and their roles in soybean domestication. *Plant J* **90**, 941–953 (2017).

10. Gregis, V., Sessa, A., Colombo, L. & Kater, M. *AGL24*, *SHORT VEGETATIVE PHASE*, and *APETALA1* redundantly control *AGAMOUS* during early stages of flower development in *Arabidopsis*. *Plant Cell* **18**, 1373-1382 (2006).

11. Z, J. *et al.* *GmFULa*, a *FRUITFULL* homolog, functions in the flowering and maturation of soybean. *Plant Cell Reports* **34**, 121–132 (2015).

12. Wenkel, S. *et al.* CONSTANS and the CCAAT box binding complex share a functionally important domain and interact to regulate flowering of *Arabidopsis*. *Plant Cell* **18**, 2971-2984 (2006).

13. Lee, J., Yun, J., Zhao, W., Shen, W. & Amasino, R. A methyltransferase required for proper timing of the vernalization response in *Arabidopsis*. *P Natl Acad Sci USA* **112**, 2269-2274 (2015).

14. Bastow, R. *et al.* Vernalization requires epigenetic silencing of *FLC* by histone methylation. *Nature* **427**, 164-167 (2004).

15. Seo, E. *et al.* Crosstalk between cold response and flowering in Arabidopsis is mediated through the flowering-time gene SOC1 and its upstream negative regulator FLC. *Plant Cell* **21**, 3185-3197 (2009).

16. Cui, X. *et al.* Ubiquitin-specific proteases UBP12 and UBP13 act in circadian clock and photoperiodic flowering regulation in *Arabidopsis*. *Plant Physiol* **162**, 897-906 (2013).

17. Sánchez-Lamas, M., Lorenzo, C. & Cerdán, P. Bottom-up assembly of the phytochrome network. *PLoS Genet* **12**, e1006413 (2016).

18. Hu, X. *et al.* Proteasome-mediated degradation of FRIGIDA modulates flowering time in Arabidopsis during vernalization. *Plant Cell* **26**, 4763–4781 (2014).

19. Farrona, S., Hurtado, L., Bowman, J. & Reyes, J. The *Arabidopsis thaliana SNF2* homolog *AtBRM* controls shoot development and flowering. *Development* **131**, 4965-4975 (2004).

20. Khanna, R., Kikis, E. & Quail, P. *EARLY FLOWERING* *4* functions in phytochrome B-regulated seedling de-etiolation. *Plant Physiol* **133**, 1530-1538 (2003).

21. Marcolino-Gomes, J. *et al.* Functional characterization of a putative Glycine max ELF4 in transgenic Arabidopsis and its role during flowering control. *Front Plant Sci* **8**, 618 (2017).

22. Wen, C. & Chang, C. *Arabidopsis RGL1* encodes a negative regulator of gibberellin responses. *Plant Cell* **14**, 87-100 (2002).
